# η^6^‐Metalated Aryl Iodides in Diels‐Alder Cycloaddition Reactions: Mode of Activation and Catalysis

**DOI:** 10.1002/asia.202201214

**Published:** 2022-12-29

**Authors:** Susana Portela, Israel Fernández

**Affiliations:** ^1^ Departamento de Química Orgánica I and Centro de Innovación en Química Avanzada (ORFEO-CINQA) Facultad de Ciencias Químicas Universidad Complutense de Madrid Ciudad Universitaria 28040 Madrid Spain

**Keywords:** DFT calculations, Diels-Alder, halogen bonding, metallocenes, reactivity

## Abstract

The potential application of η^6^‐metalated aryl iodides as organocatalyst has been explored by means of computational methods. It is found that the enhanced halogen bonding donor ability of these species, in comparison with their demetalated counterparts, translates into a significant acceleration of the Diels‐Alder cycloaddition reaction involving cyclohexadiene and methyl vinyl ketone. The factors behind this acceleration, the *endo‐exo* selectivity of the process and the influence of the nature of the transition metal fragment in the activity of these species are quantitatively explored in detail by means of the combination of the Activation Strain Model of reaction and the Energy Decomposition Analysis methods.

## Graphical abstract

Quantum‐chemical calculations predict that η^6^‐metalated aryl iodides can efficiently act as organocatalysts for Diels‐Alder cycloaddition reactions. This is due to the enhanced halogen bonding donor ability of the system which induces a significant increase in the asynchronicity of the process together with a reduction of the Pauli‐repulsion between the reactants.

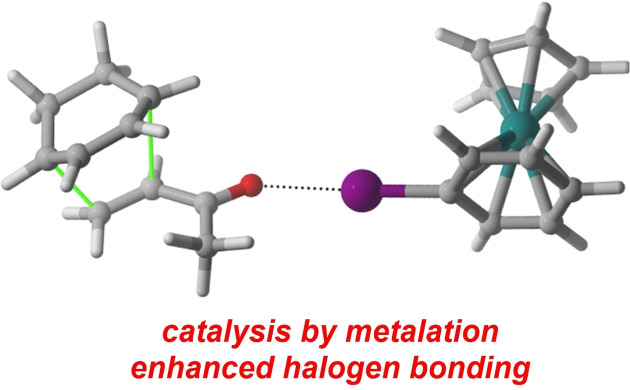

## Introduction

In the last decade, halogen bonding (XB), defined as the interaction between an electrophilic halogen substituent (called “XB donor”) and a nucleophilic Lewis base (called “XB acceptor”), has emerged as a highly useful tool in the fields of supramolecular chemistry, crystal engineering and molecular recognition.[^1^, ^2^] In addition, this highly directional interaction has been particularly useful in organocatalysis, increasing the efficiency and in many cases also the selectivity of fundamental transformations in chemistry.^[3]^ For these reasons, it is not surprising that much effort has been made to increase the strength of the XB by the judicious modification of the XB donors.^[2d]^ This includes, for instance, electron‐deficient aryl iodides or highly fluorinated alkyl iodides,^[4]^ hydrogen‐bond enhanced XB^[5]^ or even highly electrophilic iodine(III) species.^[6]^

In this regard, Kelly and Holman recently reported that the relatively low XB donor ability of iodobenzene and related aryl iodides can be significantly amplified by metalation with the [CpRu^II^]^+^ fragment, thus forming the corresponding [CpRu(ArI)]^+^ metallocene.^[7]^ The enhanced XB donor ability of these η^6^‐metalated aryl iodides is mainly ascribed to a large (ca. 8‐fold) increase in the positive potential at the σ‐hole of the iodine atom along the C−I bond induced by the transition metal fragment.

**Scheme 1:**
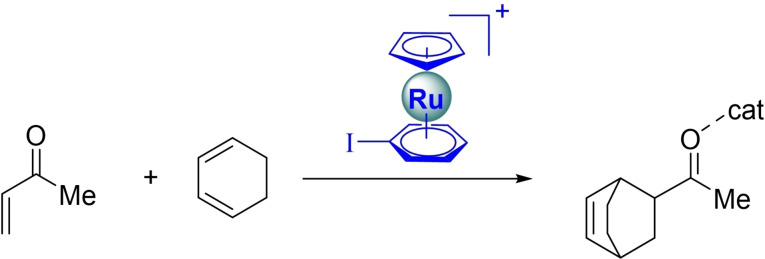
Diels‐Alder cycloaddition reaction considered herein.

Despite the firm structural and spectroscopic evidence supporting the enhancement of the XB donicity in these metallocenes,^[7]^ their potential application as organocatalysts remains unexplored so far. Based on our previous studies on the role of the halogen and chalcogen donors as catalysts in fundamental reactions in chemistry,^[8]^ herein we investigate, by means of state‐of‐the‐art computational methods, the potential catalytic activity of these η^6^‐metalated aryl iodides. To this end, the Diels‐Alder cycloaddition reaction involving cyclohexadiene and methyl vinyl ketone (**MVK**) was selected (Scheme 1).

Issues such as the influence of the η^6^‐metalated aryl iodides on both the activation barrier and the *endo/exo* selectivity of the process in comparison with the analogous uncatalyzed and demetalated aryl iodide catalyzed cycloaddition reactions as well as the impact of the nature of the transition metal fragment on the transformation shall be quantitatively analyzed in detail with the help of the combination of the Activation Strain Model (ASM)^[9]^ and Energy Decomposition Method (EDA)^[10]^ methods. This approach has been selected because it has enormously contributed to our current understanding of fundamental processes in chemistry, and, in particular, of cycloaddition reactions.[^9^, ^11^]

## Computational Details

Geometry optimizations of the molecules were performed without symmetry constraints using the Gaussian09 (RevD.01) suite of programs^[12]^ at the dispersion‐corrected B3LYP^[13]^‐D3^[14]^/def2‐SVP^[15]^ level including solvent effects (solvent=dichloromethane) with the Polarization Continuum Model (PCM) method.^[16]^ Reactants and adducts were characterized by frequency calculations and have positive definite Hessian matrices. Transition states show only one negative eigenvalue in their diagonalized force constant matrices, and their associated eigenvectors were confirmed to correspond to the motion along the reaction coordinate under consideration using the Intrinsic Reaction Coordinate (IRC) method.^[17]^ Energy refinements were carried out by means of single‐point calculations at the same DFT level using the much larger triple‐ζ basis set def2‐TZVPP.^[15]^ This level is denoted PCM(CH_2_Cl_2_)‐B3LYP‐D3/def2‐TZVPP//PCM(CH_2_Cl_2_)‐B3LYP‐D3/def2‐SVP. Natural charges were computed using the NBO6.0 method.^[18]^

### Activation Strain Model (ASM) of Reactivity and Energy Decomposition Analysis (EDA) Methods

Within the ASM method,^[9]^ also known as the distortion/interaction model,^[9c]^ the potential energy surface Δ*E*(ζ) is decomposed along the reaction coordinate, ζ, into two contributions, namely the strain Δ*E*_strain_(ζ) associated with the deformation (or distortion) required by the individual reactants during the process and the interaction Δ*E*_int_(ζ) between these increasingly deformed reactants:

$$\Delta E\left(\zeta \right)=\Delta E{}_{\mathrm{strain}}\left(\zeta \right)+\Delta E{}_{\mathrm{int}}\left(\zeta \right)$$

Within the EDA method,^[10]^ the interaction energy can be further decomposed into the following chemically meaningful terms:

$$\Delta E{}_{\mathrm{int}}\left(\zeta \right)=\Delta V{}_{\mathrm{elstat}}\left(\zeta \right)+\Delta E{}_{\mathrm{Pauli}}\left(\zeta \right)+\Delta E{}_{\mathrm{orb}}\left(\zeta \right)+\Delta E{}_{\mathrm{disp}}\left(\zeta \right)$$

The term Δ*V*_elstat_ corresponds to the classical electrostatic interaction between the unperturbed charge distributions of the deformed reactants and is usually attractive. The Pauli repulsion Δ*E*_Pauli_ comprises the destabilizing interactions between occupied orbitals and is responsible for any steric repulsion. The orbital interaction Δ*E*_orb_ accounts for bond pair formation, charge transfer (interaction between occupied orbitals on one moiety with unoccupied orbitals on the other, including HOMO‐LUMO interactions), and polarization (empty‐occupied orbital mixing on one fragment due to the presence of another fragment). Finally, the Δ*E*_disp_ term accounts for the interactions coming from dispersion forces. Moreover, the NOCV (Natural Orbital for Chemical Valence)^[19]^ extension of the EDA method has been also used to further partition the Δ*E*_orb_ term. The EDA‐NOCV approach provides pairwise energy contributions for each pair of interacting orbitals to the total bond energy.

The program package ADF^[20]^ was used for EDA calculations using the optimized PCM(CHCl_3_)‐B3LYP‐D3/def2‐SVP geometries at the same B3LYP‐D3 level in conjunction with a triple‐ζ‐quality basis set using uncontracted Slater‐type orbitals (STOs) augmented by two sets of polarization functions with a frozen‐core approximation for the core electrons.^[21]^ Auxiliary sets of s, p, d, f, and g STOs were used to fit the molecular densities and to represent the Coulomb and exchange potentials accurately in each SCF cycle.^[22]^ Scalar relativistic effects were incorporated by applying the zeroth‐order regular approximation (ZORA).^[23]^ This level of theory is denoted ZORA‐B3LYP‐D3/TZ2P//PCM(CHCl_3_)‐B3LYP‐D3/def2‐SVP.

## Results and Discussion

**Figure 1:**
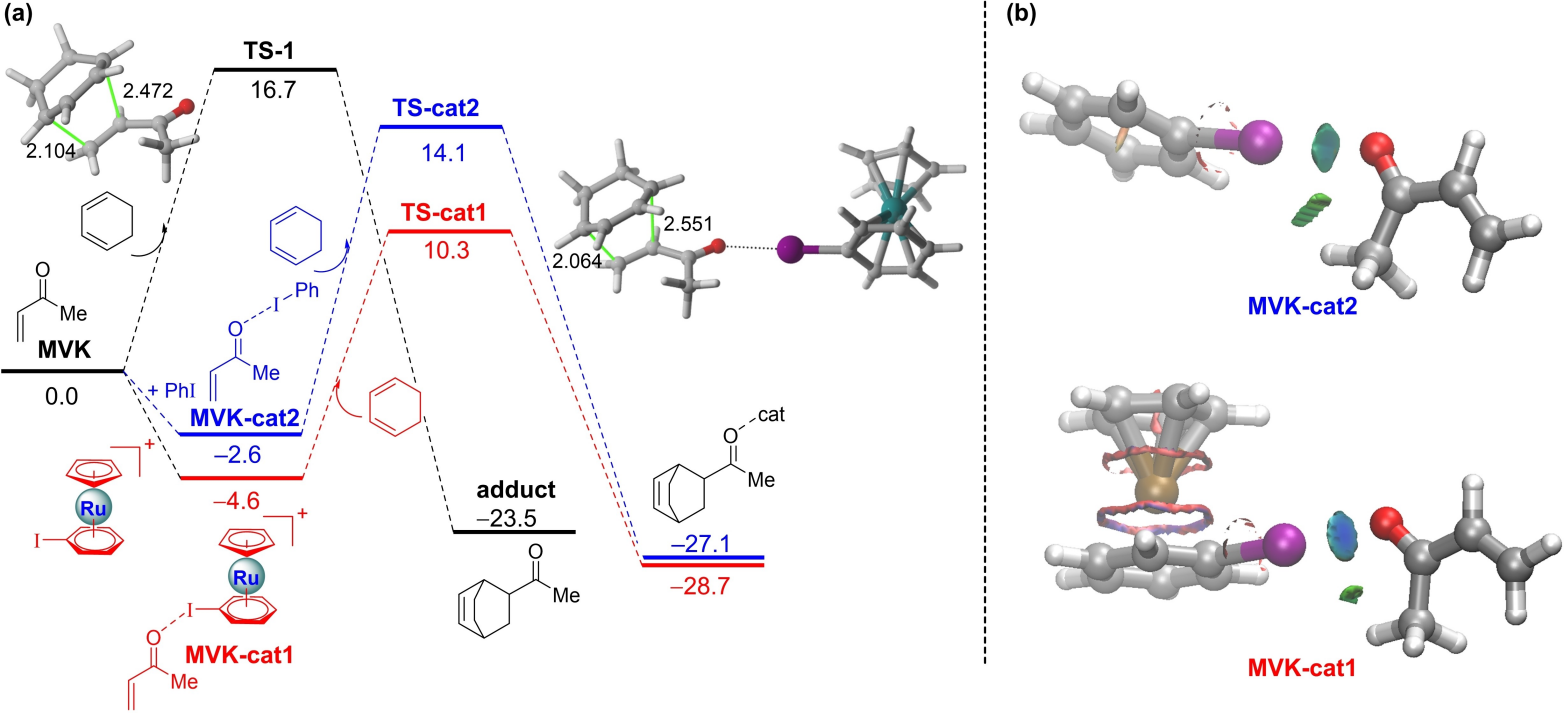
(a) Computed reaction profiles for the parent Diels‐Alder cycloaddition reactions between cyclohexadiene and **MVK** (black), the analogous process catalyzed by **cat1** (red) and **cat2** (blue). Relative energies and bond distances are given in kcal/mol and angstroms, respectively. All data have been computed at the PCM(CH_2_Cl_2_)‐B3LYP‐D3/def2‐TZVPP//PCM(CH_2_Cl_2_)‐B3LYP‐D3/def2‐SVP level. (b) Contour plots of the reduced density gradient isosurfaces (density cutoff=0.045 au) for the **MVK‐cat1** and **MVK‐cat2** complexes. The greenish surfaces indicate attractive non‐covalent interactions.

The computed reaction profiles for the uncatalyzed (*endo*) Diels‐Alder reaction involving **MVK** and cyclohexadiene and the analogous reactions catalyzed by [CpRu(PhI)]^+^ (**cat1**) and iodobenzene (**cat2**) are shown in Figure 1a. As clearly seen, our calculations indicate that in all cases the cycloaddition proceeds in a concerted yet asynchronous manner through the corresponding six‐membered transition state (**TS**), leading to the exothermic formation of the corresponding cycloadduct. As expected, the catalyzed reactions involve the initial activation of the dienophile, thus forming the halogen bonding complexes **MVK‐cat**. From the data in Figure 1a, it becomes evident that the activation of the dienophile is greater for the process mediated by the metallocene catalyst in view of the higher exothermicity of the formation of **MVK‐cat1** in comparison with **MVK‐cat2** (ΔΔE=2.0 kcal/mol), which is consistent with the enhanced XB donor ability of **cat1**.^[7]^ As a consequence, the [CpRu(PhI)]^+^‐mediated process becomes much more favored than the analogous uncatalyzed or PhI‐catalyzed reactions along the entire reaction coordinate. Indeed, whereas the barrier for the cycloaddition mediated by the low XB donor iodobenzene is nearly identical to that computed for the parent reaction, the **cat1**‐mediated reaction proceeds with a lower barrier (ΔΔE^≠^≈2 kcal/mol) in a more exothermic process (ΔΔE_R_≈−5 kcal/mol),^[24]^ which suggests that the η^6^‐metallated aryl iodide can indeed be efficiently used as an organocatalyst for this cycloaddition reaction.

Before exploring the factors leading to the acceleration of the cycloaddition induced by the metallocene catalyst, we first investigated the nature of the interaction between the catalyst and the dienophile in the **MVK‐cat** complexes. In both cases, according to the NCIPLOT method,^[25]^ there exists a clear noncovalent attractive interaction (greenish surface) between the iodine atom of the catalyst and the carbonyl oxygen atom of the **MVK** (Figure 1b), which confirms the occurrence of the halogen bonding. Despite that, the computed C=O⋅⋅⋅I distance is much shorter in the **MVK‐cat1** complex (2.742 Å) than in **MVK‐cat2** (3.058 Å), which indicates that the halogen bond strength is significantly stronger in the former species as a result of its enhanced XB donor ability.

To further quantitatively understand the enhanced halogen bond in the metallocene complex, the Energy Decomposition Analysis (EDA) method was applied next. As shown in Table 1, the instantaneous interaction energy (ΔE_int_) between **MVK** and **cat1** is significantly stronger (ΔΔE_int_=6.3 kcal/mol) in the **MVK‐cat1** complex than that computed for the **MVK‐cat2**. This is mainly due to both stronger electrostatic interactions (ΔΔV_elstat_=8.4 kcal/mol), and to a lesser extent, also to stronger orbital interactions (ΔΔE_orb_=3.0 kcal/mol, dominated by the LP(O)→σ*(I−C) interaction according to the NOCV method). This finding is fully consistent with the significant increase in the positive potential at the σ‐hole of the iodine atom reported by Kelly and Holman,^[7]^ and confirms the predominant electrostatic nature of these and related halogen bonding complexes.[^1^, ^8^]

**Table 1:** Results of the EDA (energy values in kcal/mol) computed for the **MVK‐cat** complexes.^[a]^

Compound	ΔE_int_	ΔE_Pauli_	ΔV_elstat_	ΔE_orb_	ΔE_disp_
**MVK‐cat1**	−9.6	13.1	−14.4	−6.1	−2.2
**MVK‐cat2**	−3.3	8.0	−6.0	−3.1	−2.1

[a] All data have been computed at the ZORA‐B3LYP‐D3/TZ2P//PCM(CH_2_Cl_2_)‐B3LYP‐D3/def2‐SVP level.

**Figure 2:**
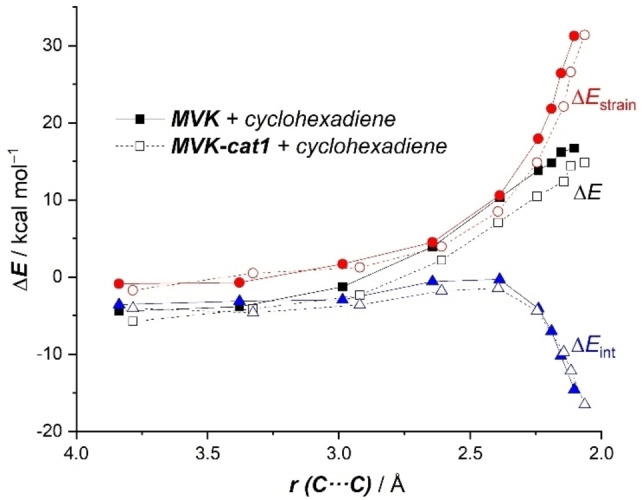
Comparative activation strain analyses of the Diels‐Alder cycloadditions reactions between cyclohexadiene and **MVK** (uncatalyzed, solid lines) and **MVK‐cat1** (dashes lines) projected onto the shorter C⋅⋅⋅C bond‐forming distance. All data have been computed at the PCM(CH_2_Cl_2_)‐B3LYP‐D3/def2‐TZVPP//PCM(CH_2_Cl_2_)‐B3LYP‐D3/def2‐SVP level.

Once the nature of the halogen bonding in the initial activated complexes has been analyzed, we then explored the factors controlling the predicted acceleration for the cycloaddition mediated by the η^6^‐metallated phenyl iodide **cat1**. To this end, the Activation Strain Model (ASM) of reactivity was then applied to compare the uncatalyzed and the **cat1**‐catalyzed reactions. Figure 2 shows the corresponding activation strain diagrams (ASDs) for both processes from the initial stages of the reaction up to the corresponding transition states and projected onto the shorter C⋅⋅⋅C bond‐forming distance.^[26]^ Although both transformations exhibit similar ASDs, from the data in Figure 2 it becomes clear that the catalyzed reaction benefits from both a less destabilizing strain energy (measured by the ΔE_strain_ term) and a stronger interaction between the deformed reactants (measured by the ΔE_int_ term) along practically the entire reaction coordinate. Similar to related catalyzed Diels‐Alder reactions,[^8c^, ^8d^, ^27^] the trend in ΔE_strain_ can be ascribed to the extent of the asynchronicity of the cycloaddition, which is markedly higher in the catalyzed reaction according to the higher difference between the newly forming C⋅⋅⋅C bond lengths in the respective transition states (Δr_C⋅⋅⋅C_=0.368 Å vs 0.487 Å for the uncatalyzed and **cat1**‐catalyzed reactions, respectively, see also Figure 1a). Thus, a higher asynchronicity implies that the corresponding transition state is reached earlier and consequently, the energy penalty to adopt the transition state geometry (i. e. the activation strain energy) is lower.^[28]^

**Figure 3:**
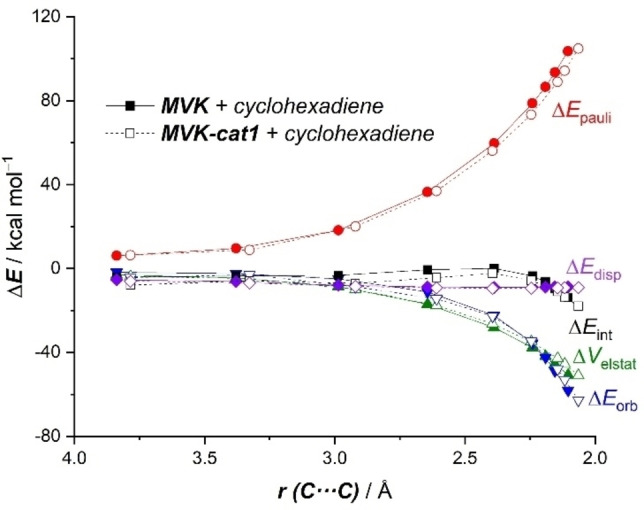
Comparative activation strain analyses of the Diels‐Alder cycloaddition reactions between cyclohexadiene and **MVK** (uncatalyzed, solid lines) and **MVK‐cat1** (dotted lines) projected onto the shorter C⋅⋅⋅C bond‐forming distance. All data have been computed at the ZORA‐B3LYP‐D3/TZ2P//PCM(CH_2_Cl_2_)‐B3LYP‐D3/def2‐SVP level.

Reasons behind the computed stronger interaction between the deformed reactants for the metallocene‐mediated cycloaddition might be initially traced to the stabilization of the π*‐LUMO of the dienophile upon binding to the catalyst, following the traditional LUMO‐lowering rationalization in catalysis.^[29]^ Indeed, our calculations indicate that the LUMO of **MVK‐cat1** is stabilized with respect to the parent **MVK** (−2.20 eV vs −1.76 eV, respectively). However, we recently reported that in many instances this LUMO‐lowering concept is not the actual factor responsible for the acceleration promoted by different types of catalysts in Diels‐Alder reactions but a significant reduction of the Pauli‐repulsion between the key occupied π‐molecular orbitals of the reactants along the entire coordinate.[^27^, ^30^] To check whether this Pauli‐repulsion lowering concept is also the key factor behind the computed enhanced interaction for the **cat1**‐mediated cycloaddition, the Energy Decomposition Analysis (EDA) method was applied next. As depicted in Figure 3, which graphically shows the evolution of the EDA contributors once again for both the uncatalyzed and catalyzed reactions from the initial stages of the reaction up to the corresponding transition states, it becomes clear that the main attractive terms (ΔV_elstat_ and ΔE_orb_) favor the uncatalyzed reaction. For instance, at the same consistent C⋅⋅⋅C bond‐forming distance of 2.15 Å,^[31]^ the difference in these terms is ΔΔV_elstat_=3.8 kcal/mol and ΔΔE_orb_=2.1 kcal/mol, favoring the uncatalyzed reaction, which indicates that neither the electrostatic attractions nor the orbital interactions (despite the more favorable HOMO(diene)‐LUMO(dienophile) gap) are responsible for the stronger interaction computed for the metallocene‐catalyzed reaction. Instead, this process benefits from a less destabilizing Pauli‐repulsion along the entire reaction coordinate (ΔΔE_Pauli_=2.1 kcal/mol at 2.15 Å), which confirms that the Pauli‐repulsion concept^[30]^ also applies in this particular catalyzed reaction. This substantial reduction in the Pauli repulsion is mainly due to the polarization induced by the catalyst on the reactive C=C bond of the dienophile away from the incoming diene. This is reflected in a noticeable depopulation of the terminal C=CH_2_ carbon atom as confirmed by the computed lower natural charge of this atom in the initial **MVK‐cat1** complex (−0.302e vs −0.321e in the parent **MVK**).

### Endo‐exo selectivity

In agreement with previous reports,[^6^, ^8b^] our calculations confirm that the parent cycloaddition reaction between **MVK** and cyclohexadiene is *endo*‐selective (ΔΔE^≠^_endo‐exo_=2.7 kcal/mol; ΔΔG^≠^_endo‐exo_=2.6 kcal/mol). Similarly, the *endo*‐approach is also favored in the analogous cycloaddition mediated by the metallocene catalyst **cat1** from both kinetic (ΔΔE^≠^_endo‐exo_=3.0 kcal/mol; ΔΔG^≠^_endo‐exo_=4.0 kcal/mol) and thermodynamic (ΔΔE_endo‐exo_=1.1 kcal/mol; ΔΔG_endo‐exo_=1.4 kcal/mol) points of view.

**Figure 4:**
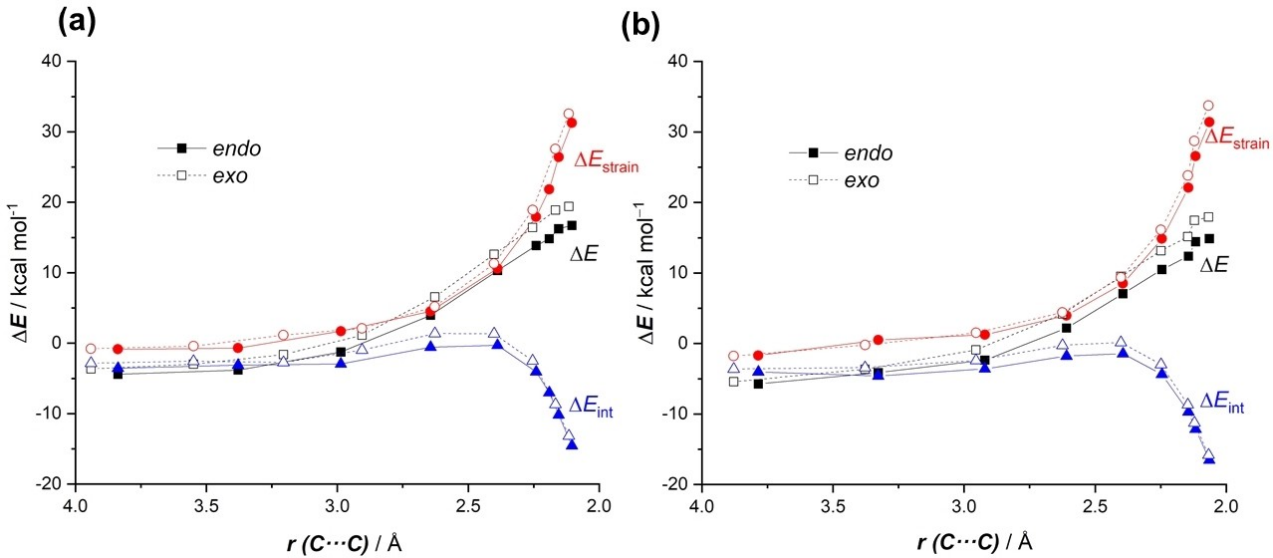
Comparative activation strain analyses for the *endo/exo* approaches of the Diels‐Alder cycloadditions reactions between cyclohexadiene and **MVK** (a) and the analogous process mediated by **cat1** (b), projected onto the shorter C⋅⋅⋅C bond‐forming distance. All data have been computed at the PCM(CH_2_Cl_2_)‐B3LYP‐D3/def2‐TZVPP//PCM(CH_2_Cl_2_)‐B3LYP‐D3/def2‐SVP level.

The reasons behind the *endo*‐preference in both processes were then analyzed by means of the ASM approach. Figure 4 shows the corresponding ASDs for both the *endo* and *exo* approaches of both cycloadditions, again from the separate reactants up to the respective transition states. The uncatalyzed and catalyzed transformations exhibit rather similar ASDs in the sense that the interaction energy between the reactants is clearly more stabilizing at the beginning of the process for the *endo*‐approach and becomes only slightly stronger at the transition state region. This indicates that the strain energy constitutes the decisive factor leading to the observed *endo*‐selectivity, particularly at the proximities of the transition states, where the *endo*‐approach benefits from a less destabilizing strain energy (measured by the ΔE_strain_ term). This is similar to the parent cycloaddition reaction between cyclopentadiene and maleic anhydride where the *endo*‐selectivity mainly derives exclusively from the distortion energy,^[32]^ which suggests that the metallocene catalyst has an almost negligible influence on the inherent *endo*‐selectivity of the process.

### Tuning the activity of the η^6^‐metallated aryl iodide catalyst

**Figure 5:**
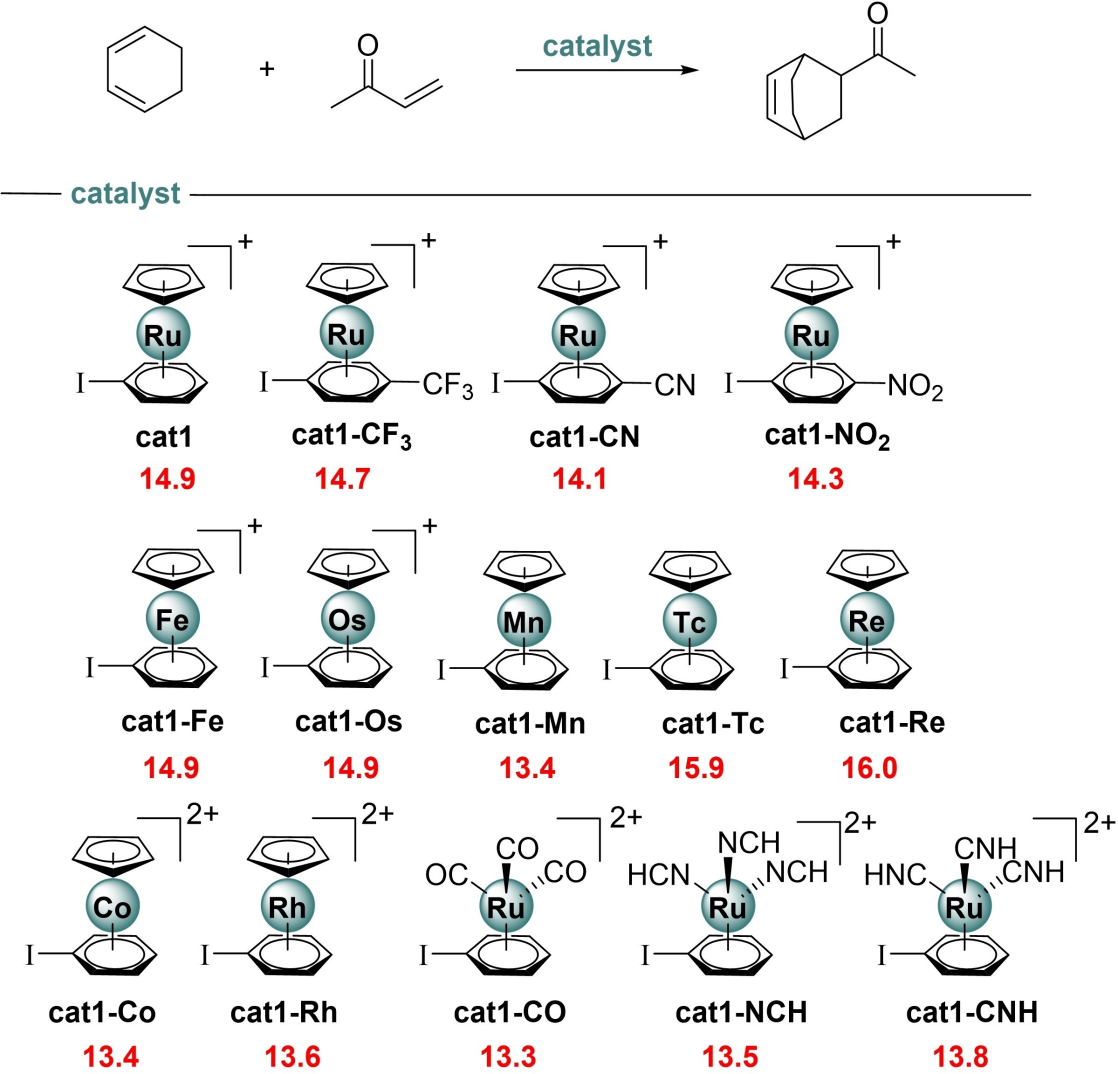
Computed activation barriers (ΔE^≠^, in kcal/mol) for the catalyzed cycloaddition reactions involving cyclohexadiene and methyl vinyl ketone. ΔE^≠^ computed as ΔE^≠^=E(**TS**) – E(cyclohexadiene) – E(**MVK‐cat** complex). All data have been computed at the PCM(CH_2_Cl_2_)‐B3LYP‐D3/def2‐TZVPP//PCM(CH_2_Cl_2_)‐B3LYP‐D3/def2‐SVP level.

Aiming at further enhancing the activity (i. e. further reducing the barrier) of the parent η^6^‐metalated aryl iodide **cat1**, we finally explored the influence of the transition metal moiety on the same Diels‐Alder reaction involving **MVK** and cyclohexadiene (see Figure 5).

The first obvious way to increase the XB donicity of the metalated aryl iodide is by means of the introduction of electron‐withdrawing groups (EWGs) in the aryl ring, as done previously for non‐metalated systems.^[4]^ Not surprisingly, the presence of EWGs such as CF_3_, CN or NO_2_ groups at the *para* position of the aryl iodide leads to a slight yet noticeable reduction of the activation barrier of the cycloaddition.

We also explored the influence of the transition metal fragment on the process with the PhI ligand unaltered. First, we modified the nature of the transition metal and found that no change in the barrier is observed when using the group 8 [CpM^II^(PhI)]^+^ analogues (M=Fe, Os) as catalysts. At variance, the barrier is significantly reduced for the cycloaddition mediated by the isoelectronic d^6^‐neutral [CpMn^I^(PhI)] complex^[33]^ **cat1‐Mn** (ΔΔE=1.5 kcal/mol with respect to **cat1**), whereas the activity of its heavier group 7 counterparts (**cat1‐Tc** and **cat1‐Re**) is comparatively lower. A similar reduction in the barrier is found for the isolectronic‐d^6^ group 9 [CpM^III^(PhI)]^2+^ complexes **cat1‐Co** (M=Co) and **cat1‐Rh** (M=Rh).^[34]^ Finally, we replaced the cyclopentadienyl ligand with strong π‐acceptor ligands to further enhance the electron‐withdrawing nature of the transition metal fragment. To our delight, the replacement of the six‐electron donor Cp ligand by three two‐electron donor CO, nitrile (NCH) or isonitrile (CNH) ligands leads in all cases to a noticeable reduction of the barrier. This initial screening of the nature of the catalyst clearly indicates that the activity of these η^6^‐metalated aryl iodides as organocatalysts can be efficiently tuned by rationally modifying the nature of the transition metal fragment.

As mentioned above, the activity of the catalyst is directly related to the activation of the dienophile, which can be estimated by the interaction between **MVK** and the catalyst in the corresponding **MVK‐cat** complex. To further support this finding, we applied the EDA method to representative initial complexes of the previous screening (Table 2). As expected, the instantaneous interaction energy (ΔE_int_) becomes only slightly stronger for the *p*‐substituted systems involving **cat1‐CF_3_**, **cat1‐CN** and **cat1‐NO_2_** which results in a slight decrease in the computed barrier. At variance, rather similar (ΔE_int_) values were found for the group 8 **MVK‐cat1** complexes, which translates into the computed nearly identical barriers for the cycloaddition reactions involving these catalysts. In contrast, the computed interaction is clearly much stronger for the Co(III) species **MVK‐cat1‐Co** and those complexes having strong acceptor ligands (**MVK‐cat1‐CO**, **MVK‐cat1‐NCH** and **MVK‐cat1‐CNH**), which exhibit the lowest activation barriers. Therefore, our calculations allow us to conclude that the catalytic activity of the metallocene catalyst directly correlates with its XB donor ability.

**Table 2:** Results of the EDA (energy values in kcal/mol) computed for the **MVK‐cat** complexes.^[a]^

Compound	ΔE_int_	ΔE_Pauli_	ΔV_elstat_	ΔE_orb_	ΔE_disp_
**MVK‐cat1**	−9.6	13.1	−14.4	−6.1	−2.2
**MVK‐cat1‐CF_3_**	−12.5	14.2	−15.6	−9.0	−2.2
**MVK‐cat1‐CN**	−11.2	14.5	−15.9	−7.7	−2.2
**MVK‐cat1‐NO_2_**	−12.9	14.9	−15.3	−10.2	−2.3
**MVK‐cat1‐Fe**	−10.4	13.3	−14.8	−6.8	−2.2
**MVK‐cat1‐Os**	−8.2	13.4	−14.6	−4.9	−2.2
**MVK‐cat1‐Co**	−24.7	19.8	−24.9	−17.5	−2.2
**MVK‐cat1‐CO**	−25.2	22.5	−25.8	−19.5	−2.4
**MVK‐cat1‐NCH**	−17.6	17.9	−19.3	−13.6	−2.6
**MVK‐cat1‐CNH**	−16.3	18.3	−19.8	−12.2	−2.6

[a] All data have been computed at the ZORA‐B3LYP‐D3/TZ2P//PCM(CH_2_Cl_2_)‐B3LYP‐D3/def2‐SVP level.

We finally applied the ASM‐EDA approach to understand the origin of the predicted further acceleration in the cycloaddition involving the more active catalysts commented above. To this end, we compared the parent **cat1**‐catalyzed reaction (ΔE^≠^=14.9 kcal/mol) with the analogous process mediated by **cat1‐NCH** (having 3 nitriles as ligands directly attached to the transition metal, ΔE^≠^=13.5 kcal/mol), as a representative system.

**Figure 6:**
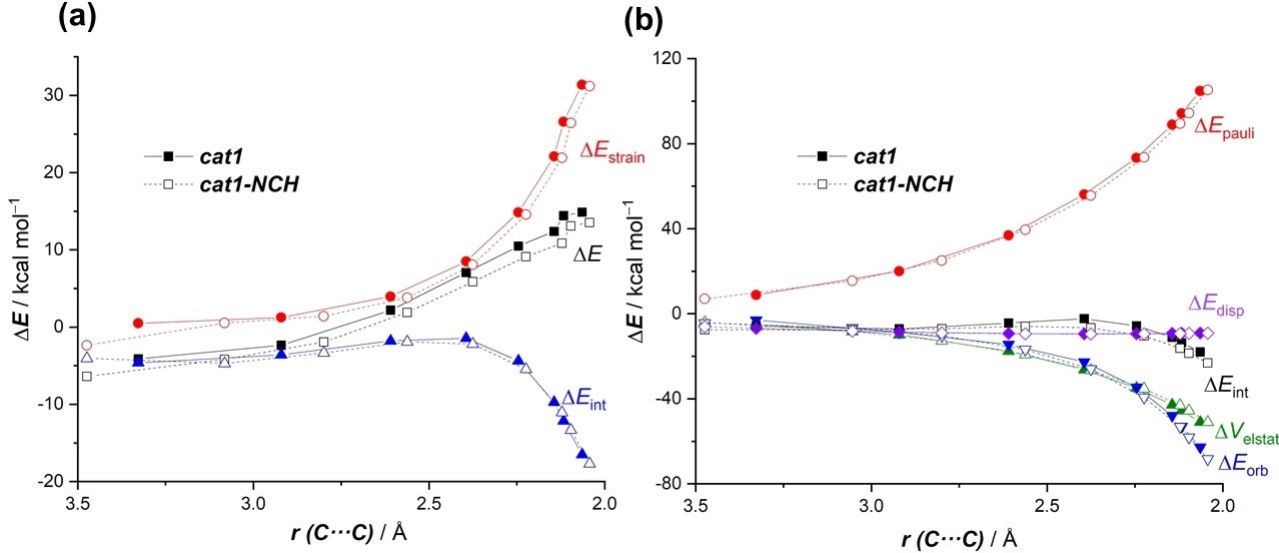
Comparative (a) activation strain analyses (PCM(CH_2_Cl_2_)‐B3LYP‐D3/def2‐TZVPP//PCM(CH_2_Cl_2_)‐B3LYP‐D3/def2‐SVP level) and (b) Energy Decomposition Analysis (ZORA‐B3LYP‐D3/TZ2P//PCM(CH_2_Cl_2_)‐B3LYP‐D3/def2‐SVP level) of the Diels‐Alder cycloadditions reactions between cyclohexadiene and **MVK‐cat1** (solid lines) and **MVK‐cat1‐NCH** (dashes lines) projected onto the shorter C⋅⋅⋅C bond‐forming distance.

As graphically shown in Figure 6a, the lower barrier computed for the cycloaddition catalyzed by **cat1‐NCH** is due to a less destabilizing strain energy along the entire coordinate and, to a lesser extent, to a stronger interaction between the deformed reactants. Once again, the less destabilizing ΔE_strain_ is ascribed to a further increase in the asynchronicity of the reaction as confirmed by the computed C⋅⋅⋅C bond‐forming distances in the corresponding transition state: 2.043 and 2.606 Å, which is translated into Δr_C⋅⋅⋅C_=0.563 Å (>Δr_C⋅⋅⋅C_=0.487 Å for the process mediated by **cat1**). The slightly stronger interaction computed for this cycloaddition is due, according to the EDA method (Figure 6b), once again mainly to a reduction of the Pauli repulsion between the key π‐molecular orbitals of both reactants and also, albeit to a much lesser extent, to stronger orbital interactions at the transition state region. Not surprisingly, the further reduction of the Pauli repulsion is again directly related to a higher depopulation of the reactive C=C of the dienophile, which is confirmed by an even lower natural charge at the terminal C=CH_2_ carbon atom in the corresponding **MVK‐cat1‐NCH** complex (−0.293e vs −0.302e in **MVK‐cat1**).

## Conclusion

From the computational study reported herein, it is predicted that η^6^‐metalated aryl iodides can be used as organocatalysts for the Diels‐Alder cycloaddition reaction involving cyclohexadiene and methyl vinyl ketone. These species induce a noticeable acceleration of the process by reducing the corresponding activation barrier up to 3–4 kcal/mol with respect to the parent uncatalyzed reaction or the analogous process involving the corresponding demetalated aryl iodide without modifying the innate *endo*‐selectivity of the transformation. The metallocene catalyst binds the carbonyl group of the dienophile through a halogen bond, whose strength can be efficiently modulated by increasing the acceptor nature of the transition metal fragment. This non‐covalent interaction induces a significant depopulation of the key π‐C=C molecular orbital of the dienophile which translates into a reduction of the Pauli repulsion between the reactants along the entire reaction coordinate. As a result, the interaction energy between the reactants becomes stronger than that in the parent uncatalyzed reaction. In addition, the catalyst also increases the asynchronicity of the reaction which leads to a less destabilizing strain energy. Therefore, the catalytic activity of these η^6^‐metalated aryl iodides, which can be efficiently tuned, finds its origin in both a higher asynchronicity of the process and a reduction of the Pauli repulsion between the deformed reactants.

## Conflict of interest

The authors declare no conflict of interest.

1

## Supporting information

As a service to our authors and readers, this journal provides supporting information supplied by the authors. Such materials are peer reviewed and may be re‐organized for online delivery, but are not copy‐edited or typeset. Technical support issues arising from supporting information (other than missing files) should be addressed to the authors.

Supporting Information

## Data Availability

The data that support the findings of this study are available in the supplementary material of this article.
