# Supplementary material for: η6‐Metalated Aryl Iodides in Diels‐Alder Cycloaddition Reactions: Mode of Activation and Catalysis
Source: Chem Asian J. 2022 Dec 29;18(3):e202201214. doi: 10.1002/asia.202201214 (PMC10108214; doi:10.1002/asia.202201214)
Supplement: Supplementary file 1 — Supporting Information [file ASIA-18-0-s001.pdf]

CHEMISTRY
AN ASIAN JOURNAL

Supporting Information

η^6-Metalated Aryl Iodides in Diels-Alder Cycloaddition Reactions: Mode of Activation and Catalysis

Susana Portela and Israel Fernández*This manuscript is part of a joint special collection on Mechanisms and Selectivities of Organic Reactions – In Celebration of Prof. Kendall N. Houk's 80th birthday.© 2022 The Authors. Chemistry - An Asian Journal published by Wiley-VCH GmbH. This is an open access article under the terms of the Creative Commons Attribution License, which permits use, distribution and reproduction in any medium, provided the original work is properly cited.

Contents:

1. Cartesian coordinates and energies………………………………………………….....

Cartesian coordinates (in Å) and total energies (in a.u., noncorrected ZVPE included) of all the stationary points discussed in the text. All calculations have been performed at the PCM(CH_2Cl_2)-B3LYP-D3/def2-SVP level. ***SP*** denotes values computed at the PCM(CH_2Cl_2)-B3LYP-D3/def2-SVP//PCM(CH_2Cl_2)B3LYP-D3/def2-TZVPP.

MVK
E **=** -230.987048
H **=** -230.980427
G **=** -231.015674
$\boldsymbol{N_{imag}}$ **=** 0
SP **=** -231.3454216

C	-0.871942000	-0.637112000	0.000430000
H	-0.985781000	-1.726429000	0.001097000
C	-1.943763000	0.167549000	-0.000272000
H	-2.958070000	-0.241331000	-0.000231000
H	-1.855746000	1.257673000	-0.001041000
C	0.548173000	-0.186953000	0.000039000
C	0.850921000	1.297997000	0.000189000
H	0.413947000	1.784677000	-0.886434000
H	0.414663000	1.784313000	0.887347000
H	1.938716000	1.443186000	-0.000319000
O	1.441492000	-1.018872000	-0.000342000

Cyclohexadiene
E **=** -233.140630
H **=** -233.134501
G **=** -233.168875
$\boldsymbol{N_{imag}}$ **=** 0
SP **=** -233.5200151

C	-0.109699000	-1.426747000	0.065121000
C	1.195409000	-0.729188000	-0.240747000
C	1.194347000	0.730841000	0.240711000
C	-0.111694000	1.426610000	-0.065081000
C	-1.262196000	0.726100000	-0.105651000
C	-1.261180000	-0.727845000	0.105616000
H	1.350198000	0.759743000	1.339374000
H	2.046361000	-1.274685000	0.196936000
H	1.351311000	-0.758109000	-1.339369000
H	-0.112120000	-2.514741000	0.184956000
H	-0.115615000	2.514590000	-0.184916000
H	-2.218160000	1.231530000	-0.272934000
H	-2.216422000	-1.234608000	0.272967000
H	2.044527000	1.277654000	-0.196829000

Cat1
E **=** -817.329113
H **=** -817.316940
G **=** -817.369089
$\boldsymbol{N_{imag}}$ **=** 0
SP **=** -818.0080105

C	0.908721000	0.807512000	-0.000030000
C	0.304694000	1.181083000	-1.234011000
C	-0.900079000	1.935659000	-1.228007000
C	-1.504088000	2.309981000	0.000982000
C	-0.900305000	1.933240000	1.230076000
C	0.304948000	1.179698000	1.234950000
H	-1.383676000	2.182100000	-2.174679000
H	-2.455430000	2.843957000	0.001766000
H	-1.384339000	2.178094000	2.176886000

H	0.741730000	0.858275000	2.181241000
I	2.701881000	-0.324863000	-0.000028000
H	0.740798000	0.860799000	-2.181023000
C	-3.202749000	-0.843740000	-0.064454000
C	-2.537345000	-1.249157000	1.136608000
C	-2.431699000	-1.311575000	-1.177050000
H	-4.130088000	-0.275671000	-0.122335000
C	-1.355861000	-1.969546000	0.766820000
H	-2.869684000	-1.043612000	2.153204000
C	-1.290792000	-2.008498000	-0.662413000
H	-2.670715000	-1.164182000	-2.229434000
H	-0.628868000	-2.403551000	1.452018000
H	-0.505415000	-2.477317000	-1.253941000
Ru	-1.205151000	0.087975000	-0.000523000

Cat2
***E* =** -529.192228
***H* =** -529.185422
***G* =** -529.223977
N_{imag} = 0
***SP* =** -529.5460196

C	-0.570582000	0.000029000	0.000005000
C	-1.257953000	-1.218743000	0.000006000
C	-2.657557000	-1.209855000	0.000002000
C	-3.359289000	-0.000019000	-0.000002000
C	-2.657593000	1.209830000	0.000002000
C	-1.257983000	1.218762000	0.000006000
H	-3.197821000	-2.159920000	-0.000016000
H	-4.451815000	-0.000026000	-0.000022000
H	-3.197885000	2.159880000	-0.000019000
H	-0.715782000	2.166100000	0.000003000
I	1.563109000	0.000000000	-0.000001000
H	-0.715725000	-2.166065000	-0.000002000

Cat1-CF_3
***E* =** -1154.111566
***H* =** -1154.095778
***G* =** -1154.156370
N_{imag} = 0
***SP* =** -1155.1981002

C	1.159878000	-0.868898000	0.000010000
C	0.454805000	-0.957410000	1.233912000
C	-0.953761000	-1.123485000	1.234745000
C	-1.659359000	-1.184630000	0.000022000
C	-0.953757000	-1.123574000	-1.234705000
C	0.454804000	-0.957491000	-1.233889000
H	-1.498005000	-1.140359000	2.179823000
H	-1.498004000	-1.140516000	-2.179782000
H	0.983680000	-0.852579000	-2.181806000
I	3.256866000	-0.585855000	0.000003000
H	0.983674000	-0.852425000	2.181825000
C	-1.903796000	2.348460000	-0.000028000
C	-1.067973000	2.447708000	-1.158264000
C	-1.068026000	2.447642000	1.158257000
H	-2.985060000	2.215770000	-0.000048000
C	0.284762000	2.610692000	-0.715478000
H	-1.401935000	2.403166000	-2.194122000
C	0.284734000	2.610647000	0.715548000
H	-1.402044000	2.403017000	2.194094000
H	1.161513000	2.705639000	-1.355090000
H	1.161452000	2.705568000	1.355207000

Ru	-0.473421000	0.670636000	-0.000025000
C	-3.169431000	-1.310014000	0.000015000
F	-3.702275000	-0.730891000	-1.085368000
F	-3.536110000	-2.598151000	-0.000223000
F	-3.702269000	-0.731273000	1.085591000

Cat1-NO_2
***E* =** -1021.664657
***H* =** -1021.649814
***G* =** -1021.708465
N_{imag} = 0
***SP* =** -1022.5876026

C	1.047911000	-0.851313000	0.003534000
C	0.354531000	-0.998915000	1.239879000
C	-1.032297000	-1.290914000	1.246851000
C	-1.706746000	-1.425439000	0.005569000
C	-1.032607000	-1.299335000	-1.236851000
C	0.354298000	-1.005773000	-1.232013000
H	-1.589352000	-1.367722000	2.179942000
H	-1.589306000	-1.384901000	-2.169415000
H	0.873299000	-0.856862000	-2.179614000
I	3.111932000	-0.390217000	0.002188000
H	0.873890000	-0.845088000	2.186520000
C	-2.278050000	2.094468000	0.095390000
C	-1.553535000	2.233839000	-1.131816000
C	-1.359987000	2.287293000	1.176378000
H	-3.339566000	1.867306000	0.191041000
C	-0.186739000	2.516598000	-0.808496000
H	-1.968704000	2.138117000	-2.134320000
C	-0.067026000	2.549019000	0.617479000
H	-1.601153000	2.236757000	2.237419000
H	0.622616000	2.667538000	-1.521869000
H	0.849635000	2.728821000	1.178018000
Ru	-0.715163000	0.540371000	-0.002000000
N	-3.177592000	-1.645537000	0.006362000
O	-3.715984000	-1.784923000	-1.075286000
O	-3.733009000	-1.667226000	1.088328000

Cat1-CN
***E* =** -909.493697
***H* =** -909.479661
***G* =** -909.535534
N_{imag} = 0
***SP* =** -910.2758474

C	0.949158000	-0.848201000	-0.000050000
C	0.272238000	-1.063196000	1.234097000
C	-1.079813000	-1.488320000	1.238568000
C	-1.764530000	-1.702527000	0.000244000
C	-1.080016000	-1.488524000	-1.238226000
C	0.272032000	-1.063403000	-1.234045000
H	-1.608137000	-1.614634000	2.184287000
H	-1.608485000	-1.615018000	-2.183839000
H	0.772985000	-0.861023000	-2.181589000
I	2.960287000	-0.194454000	-0.000292000
H	0.773346000	-0.860645000	2.181524000
C	-2.623356000	1.785962000	0.000056000
C	-1.815904000	2.020725000	-1.158586000
C	-1.815970000	2.020586000	1.158773000
H	-3.666393000	1.470715000	0.000009000
C	-0.508121000	2.402394000	-0.715634000
H	-2.138232000	1.922216000	-2.194393000

C	-0.508161000	2.402311000	0.715942000
H	-2.138356000	1.921938000	2.194550000
H	0.341797000	2.640345000	-1.354277000
H	0.341718000	2.640186000	1.354664000
Ru	-0.931683000	0.367348000	0.000042000
C	-3.150505000	-2.087333000	0.000382000
N	-4.264820000	-2.406315000	0.000503000

Cat1-Mn
***E* =** -1873.413262
***H* =** -1873.401310
***G* =** -1873.451927
N_{imag} = 0
***SP* =** -1874.2145302

C	0.632288000	0.829057000	-0.000009000
C	0.010120000	1.143568000	-1.238336000
C	-1.259218000	1.783099000	-1.230474000
C	-1.893225000	2.099561000	0.000051000
C	-1.259249000	1.782985000	1.230555000
C	0.010105000	1.143449000	1.238351000
H	-1.780086000	1.953368000	-2.174407000
H	-2.904083000	2.510327000	0.000017000
H	-1.780107000	1.953161000	2.174511000
H	0.463104000	0.832292000	2.180272000
I	2.501231000	-0.214982000	0.000001000
H	0.463178000	0.832566000	-2.180282000
C	-3.154119000	-0.947050000	-0.000354000
C	-2.398023000	-1.310989000	1.155120000
C	-2.397454000	-1.311461000	-1.155305000
H	-4.130644000	-0.464335000	-0.000703000
C	-1.174955000	-1.902364000	0.714094000
H	-2.697011000	-1.155713000	2.191018000
C	-1.174612000	-1.902658000	-0.713438000
H	-2.695926000	-1.156604000	-2.191415000
H	-0.372858000	-2.269957000	1.353092000
H	-0.372155000	-2.270417000	-1.351888000
Mn	-1.296344000	0.087447000	-0.000072000

Cat1-Tc
***E* =** -803.389618
***H* =** -803.377264
***G* =** -803.430885
N_{imag} = 0
***SP* =** -804.063547

C	0.875723000	0.797347000	0.001276000
C	0.296863000	1.189803000	-1.242336000
C	-0.902981000	1.959342000	-1.232826000
C	-1.507489000	2.328536000	0.003651000
C	-0.909693000	1.946008000	1.239683000
C	0.291771000	1.177377000	1.246564000
H	-1.382798000	2.223068000	-2.177853000
H	-2.454616000	2.871737000	0.003798000
H	-1.393868000	2.200469000	2.185028000
H	0.738197000	0.855710000	2.189089000
I	2.703244000	-0.336650000	0.000024000
H	0.745574000	0.876274000	-2.186561000
C	-3.225155000	-0.885390000	-0.195643000
C	-2.670854000	-1.213516000	1.082018000
C	-2.356938000	-1.412634000	-1.202824000
H	-4.158523000	-0.350563000	-0.370630000
C	-1.461056000	-1.947225000	0.863713000

H	-3.109852000	-0.975380000	2.050658000
C	-1.267117000	-2.069632000	-0.548168000
H	-2.512943000	-1.350272000	-2.279573000
H	-0.813777000	-2.361002000	1.636571000
H	-0.443450000	-2.588666000	-1.037941000
Tc	-1.196844000	0.121419000	-0.002430000

Cat1-Re

***E* =** -800.858250
***H* =** -800.845761
***G* =** -800.899880
N_{imag} = 0
***SP* =** -801.5236839

C	1.081642000	0.768360000	0.000115000
C	0.504125000	1.164025000	-1.245435000
C	-0.689177000	1.948294000	-1.237383000
C	-1.286427000	2.331658000	0.000840000
C	-0.689963000	1.946123000	1.238829000
C	0.503702000	1.162194000	1.246212000
H	-1.168572000	2.212648000	-2.181938000
H	-2.227532000	2.884415000	0.000840000
H	-1.169897000	2.209234000	2.183442000
H	0.947519000	0.838668000	2.189009000
I	2.921173000	-0.355165000	-0.000010000
H	0.947936000	0.841773000	-2.188707000
C	-3.094180000	-0.873659000	-0.030244000
C	-2.408224000	-1.309276000	1.148902000
C	-2.359947000	-1.340134000	-1.167783000
H	-4.025954000	-0.309952000	-0.056994000
C	-1.250362000	-2.047714000	0.739980000
H	-2.728334000	-1.138039000	2.176029000
C	-1.220604000	-2.066903000	-0.691324000
H	-2.637094000	-1.196479000	-2.211478000
H	-0.533066000	-2.533134000	1.400721000
H	-0.476345000	-2.568918000	-1.308411000
Re	-1.017258000	0.099810000	-0.000243000

Cat1-Fe

***E* =** -1985.972184
***H* =** -1985.960404
***G* =** -1986.010982
N_{imag} = 0
***SP* =** -1986.791385

C	-0.663391000	0.810576000	-0.000882000
C	-0.027837000	1.126467000	1.227265000
C	1.233612000	1.770367000	1.221882000
C	1.866511000	2.091187000	-0.001514000
C	1.236173000	1.764067000	-1.224973000
C	-0.025606000	1.120131000	-1.229745000
H	1.750368000	1.940706000	2.166850000
H	2.872928000	2.510020000	-0.001464000
H	1.754553000	1.930514000	-2.169718000
H	-0.467057000	0.802844000	-2.174416000
I	-2.518067000	-0.212666000	0.000025000
H	-0.470427000	0.813158000	2.172733000
C	3.137645000	-0.902109000	0.191982000
C	2.559255000	-1.190354000	-1.081185000
C	2.235178000	-1.358053000	1.200026000
H	4.081103000	-0.386516000	0.363442000
C	1.299653000	-1.825582000	-0.859727000
H	2.984782000	-0.933236000	-2.049719000

C	1.099505000	-1.928499000	0.549646000
H	2.369236000	-1.250898000	2.275188000
H	0.594219000	-2.134907000	-1.629310000
H	0.213366000	-2.327357000	1.041066000
Fe	1.310397000	0.055300000	0.001821000

Cat1-Os

E **=** -813.112544
H **=** -813.100301
G **=** -813.153054
N_{imag} **=** 0
SP **=** -813.7830915

C	-1.115571000	0.786937000	-0.000178000
C	-0.512155000	1.164322000	1.235262000
C	0.686339000	1.933408000	1.230582000
C	1.285251000	2.318788000	-0.000082000
C	0.686444000	1.933334000	-1.230659000
C	-0.511628000	1.163724000	-1.235554000
H	1.170160000	2.178933000	2.176640000
H	2.232493000	2.858856000	-0.000140000
H	1.170513000	2.178434000	-2.176721000
H	-0.940289000	0.832440000	-2.181556000
I	-2.914806000	-0.341224000	-0.000005000
H	-0.941580000	0.833884000	2.181190000
C	3.053350000	-0.847269000	-0.036666000
C	2.311307000	-1.314921000	-1.171199000
C	2.370613000	-1.279747000	1.147891000
H	3.974914000	-0.268335000	-0.068749000
C	1.170816000	-2.037050000	-0.687080000
H	2.570313000	-1.153708000	-2.216443000
C	1.207515000	-2.015206000	0.745000000
H	2.682705000	-1.087078000	2.173106000
H	0.407477000	-2.514888000	-1.299233000
H	0.477325000	-2.474043000	1.409775000
Os	1.024829000	0.077137000	0.000243000

Cat1-Co

E **=** -2104.783223
H **=** -2104.771381
G **=** -2104.821657
N_{imag} **=** 0
SP **=** -2105.6230886

C	-0.721690000	0.764454000	0.001412000
C	-0.072004000	1.079368000	1.226907000
C	1.165766000	1.766865000	1.224927000
C	1.784443000	2.123311000	0.003525000
C	1.161732000	1.779483000	-1.219563000
C	-0.075090000	1.091024000	-1.222746000
H	1.678585000	1.946181000	2.171024000
H	2.774036000	2.581881000	0.004555000
H	1.670745000	1.967959000	-2.165847000
H	-0.504415000	0.766412000	-2.171449000
I	-2.567653000	-0.231676000	-0.000152000
H	-0.495938000	0.743470000	2.174717000
C	3.132560000	-0.840541000	-0.471637000
C	2.071042000	-1.449391000	-1.211235000
C	2.846027000	-0.994997000	0.920673000
H	3.978641000	-0.299453000	-0.894196000
C	1.129955000	-1.981580000	-0.276087000
H	1.969729000	-1.456430000	-2.296084000
C	1.608563000	-1.702243000	1.040906000

H	3.436617000	-0.594995000	1.744354000
H	0.182019000	-2.459096000	-0.524379000
H	1.090975000	-1.934090000	1.970914000
Co	1.337658000	0.044536000	-0.004003000

Cat1-Rh
***E* =** -832.739489
***H* =** -832.727199
***G* =** -832.778800
N_{imag} = 0
***SP* =** -833.427592

C	-0.981118000	0.787922000	0.000360000
C	-0.369142000	1.169173000	1.229796000
C	0.808243000	1.961331000	1.226544000
C	1.398836000	2.361379000	0.000630000
C	0.807439000	1.963046000	-1.225430000
C	-0.370057000	1.171106000	-1.228913000
H	1.283447000	2.219678000	2.174765000
H	2.328415000	2.932798000	0.000785000
H	1.281796000	2.222820000	-2.173682000
H	-0.791372000	0.837554000	-2.178734000
I	-2.742198000	-0.355609000	-0.000005000
H	-0.789507000	0.833960000	2.179459000
C	3.261418000	-0.748047000	-0.000960000
C	2.562813000	-1.221721000	-1.159739000
C	2.563872000	-1.219645000	1.159324000
H	4.149042000	-0.115178000	-0.001867000
C	1.435571000	-1.988235000	-0.714730000
H	2.829551000	-1.017998000	-2.196855000
C	1.436202000	-1.986908000	0.716725000
H	2.831559000	-1.014114000	2.195840000
H	0.689806000	-2.461919000	-1.353186000
H	0.691064000	-2.459417000	1.356779000
Rh	1.233516000	0.074948000	-0.000548000

Cat1-CO
***E* =** -963.513381
***H* =** -963.498477
***G* =** -963.556352
N_{imag} = 0
***SP* =** -964.3218285

C	1.044651000	-0.783918000	-0.002050000
C	0.404163000	-1.130965000	1.221207000
C	-0.772123000	-1.930698000	1.208874000
C	-1.371084000	-2.319238000	-0.012238000
C	-0.774391000	-1.905046000	-1.234619000
C	0.397556000	-1.111415000	-1.236018000
H	-1.232661000	-2.211120000	2.159437000
H	-2.283135000	-2.917956000	-0.016878000
H	-1.242880000	-2.166222000	-2.186502000
H	0.827163000	-0.789083000	-2.186373000
I	2.887586000	0.194072000	0.001471000
H	0.837823000	-0.830546000	2.176622000
Ru	-1.265521000	0.029110000	0.001006000
C	-2.647497000	0.446196000	-1.290441000
C	-2.439366000	0.576089000	1.444236000
C	-0.589499000	1.843059000	-0.139503000
O	-0.156801000	2.882833000	-0.223064000
O	-3.116973000	0.860846000	2.301461000
O	-3.448714000	0.661805000	-2.056548000

Cat1-NCH
***E* =** -903.861296
***H* =** -903.844807
***G* =** -903.905551
N_{imag} = 0
***SP* =** -904.6247912

C	0.973782000	-0.851646000	0.006964000
C	0.351343000	-1.198935000	1.224048000
C	-0.888525000	-1.922465000	1.210429000
C	-1.498363000	-2.275787000	-0.005117000
C	-0.874948000	-1.899369000	-1.240347000
C	0.334671000	-1.181261000	-1.241518000
H	-1.388262000	-2.140820000	2.156300000
H	-2.465558000	-2.780391000	-0.009768000
H	-1.375872000	-2.104997000	-2.188644000
H	0.770727000	-0.853534000	-2.186444000
I	2.802814000	0.189202000	0.000219000
H	0.790037000	-0.896931000	2.176103000
Ru	-1.161510000	-0.053100000	-0.000128000
N	-2.893633000	0.370223000	-0.979097000
N	-1.977206000	0.903687000	1.603321000
N	-0.534448000	1.787950000	-0.612800000
C	-0.164263000	2.821619000	-0.952747000
C	-2.435636000	1.429286000	2.516817000
C	-3.874652000	0.599532000	-1.532261000
H	0.189558000	3.794844000	-1.275947000
H	-2.866795000	1.920748000	3.382545000
H	-4.799946000	0.810923000	-2.057715000

Cat1-CNH
***E* =** -903.884486
***H* =** -903.868179
***G* =** -903.928233
N_{imag} = 0
***SP* =** -904.6450102

C	1.018217000	-0.841924000	0.012939000
C	0.406038000	-1.198203000	1.232587000
C	-0.803724000	-1.962039000	1.225595000
C	-1.400550000	-2.348928000	0.014212000
C	-0.793437000	-1.960303000	-1.219535000
C	0.392148000	-1.203910000	-1.227664000
H	-1.273504000	-2.221265000	2.176321000
H	-2.329909000	-2.919700000	0.013219000
H	-1.266079000	-2.224807000	-2.167376000
H	0.836225000	-0.901646000	-2.177007000
I	2.842774000	0.205292000	-0.001204000
H	0.851401000	-0.903853000	2.183689000
Ru	-1.217816000	-0.005874000	-0.001300000
C	-2.862678000	0.307019000	-0.997659000
C	-2.056364000	0.841390000	1.543867000
C	-0.606472000	1.759435000	-0.559923000
N	-0.204871000	2.796561000	-0.889673000
N	-2.544992000	1.324231000	2.478568000
N	-3.842704000	0.471987000	-1.596126000
H	0.155409000	3.695920000	-1.179855000
H	-2.970740000	1.740383000	3.296252000
H	-4.696996000	0.608298000	-2.120142000

MVK-Cat1
***E* =** -1048.326449
***H* =** -1048.306459

G **=** -1048.379416
***N*_{imag}** **=** 0
SP **=** -1049.3607627

C	-6.389116000	1.151889000	-0.052845000
H	-6.181346000	2.205045000	-0.267858000
C	-7.645139000	0.716663000	0.121725000
H	-8.493396000	1.403275000	0.053886000
H	-7.878399000	-0.329782000	0.336975000
C	-5.171803000	0.305212000	0.018345000
C	-5.305884000	-1.170686000	0.311345000
H	-5.795843000	-1.325060000	1.285727000
H	-5.935506000	-1.657506000	-0.449960000
H	-4.316128000	-1.643485000	0.322097000
O	-4.079256000	0.832805000	-0.163509000
C	0.531813000	-1.044028000	-0.053775000
C	1.245240000	-1.270600000	-1.266278000
C	2.577003000	-1.767322000	-1.228982000
C	3.206120000	-2.033888000	0.015939000
C	2.499314000	-1.805099000	1.226405000
C	1.167220000	-1.308391000	1.194022000
H	3.130041000	-1.895905000	-2.160905000
H	4.244701000	-2.366744000	0.043505000
H	2.991572000	-1.964541000	2.187206000
H	0.647654000	-1.093214000	2.129058000
I	-1.453183000	-0.273796000	-0.101787000
H	0.786776000	-1.025766000	-2.225552000
C	4.224050000	1.409614000	-0.017257000
C	3.503019000	1.629024000	1.200600000
C	3.359728000	1.742998000	-1.109320000
H	5.249204000	1.050505000	-0.098472000
C	2.193081000	2.099117000	0.860614000
H	3.883639000	1.467247000	2.208285000
C	2.104464000	2.168911000	-0.566099000
H	3.612102000	1.682963000	-2.167252000
H	1.399628000	2.351961000	1.562784000
H	1.230909000	2.480790000	-1.137444000
Ru	2.465511000	0.081102000	0.026229000

MVK-Cat2
E **=** -760.185795
H **=** -760.171081
G **=** -760.232136
***N*_{imag}** **=** 0
SP **=** -760.8956561

C	-5.145609000	-0.312116000	-0.294211000
H	-5.254775000	-1.085889000	-1.061310000
C	-6.219704000	0.255587000	0.272187000
H	-7.232392000	-0.038969000	-0.016783000
H	-6.135233000	1.029925000	1.039855000
C	-3.730376000	0.015416000	0.026199000
C	-3.433414000	1.069221000	1.069958000
H	-3.875610000	2.035017000	0.777761000
H	-3.877188000	0.787873000	2.038199000
H	-2.348478000	1.183594000	1.184564000
O	-2.837962000	-0.577729000	-0.564876000
C	2.293328000	0.000617000	-0.005578000
C	3.039415000	-1.021882000	0.593919000
C	4.424372000	-0.872829000	0.731285000
C	5.060811000	0.286104000	0.274828000
C	4.306777000	1.301518000	-0.322590000
C	2.921132000	1.164850000	-0.465889000

H	5.005680000	-1.671696000	1.199086000
H	6.142271000	0.397590000	0.384095000
H	4.795868000	2.210431000	-0.682599000
H	2.339105000	1.961718000	-0.933319000
I	0.179144000	-0.218438000	-0.219689000
H	2.549445000	-1.929446000	0.952360000

MVK-Cat1-CF_3
E **=** -1385.109624
H **=** -1385.085999
G **=** -1385.167919
N_{imag} **=** 0
SP **=** -1386 .5515181

C	7.132645000	0.403174000	-0.262164000
H	7.117552000	1.068540000	-1.131333000
C	8.274002000	0.146433000	0.392939000
H	9.218879000	0.595283000	0.074711000
H	8.314167000	-0.513513000	1.263949000
C	5.801848000	-0.150592000	0.090662000
C	5.672242000	-1.089180000	1.266720000
H	6.307018000	-1.977110000	1.119360000
H	6.013235000	-0.595566000	2.190404000
H	4.628130000	-1.403806000	1.384943000
O	4.833220000	0.173938000	-0.589711000
C	0.003612000	-0.809837000	-0.196413000
C	-0.610902000	-1.015313000	1.072507000
C	-2.010645000	-1.225848000	1.166593000
C	-2.806965000	-1.218553000	-0.013369000
C	-2.197867000	-1.042543000	-1.287843000
C	-0.797443000	-0.831283000	-1.374793000
H	-2.482214000	-1.328444000	2.144594000
H	-2.811546000	-1.006051000	-2.188782000
H	-0.345911000	-0.635422000	-2.348353000
I	2.092796000	-0.422304000	-0.327500000
H	-0.015788000	-0.960519000	1.985116000
C	-3.124921000	2.299593000	0.353662000
C	-2.464404000	2.496126000	-0.901675000
C	-2.135671000	2.350593000	1.387214000
H	-4.191936000	2.132748000	0.496876000
C	-1.066089000	2.670955000	-0.642919000
H	-2.940889000	2.506409000	-1.881107000
C	-0.863123000	2.580745000	0.770718000
H	-2.318353000	2.231631000	2.454466000
H	-0.290718000	2.832335000	-1.390888000
H	0.093619000	2.660786000	1.285458000
Ru	-1.674905000	0.668858000	0.040590000
C	-4.308014000	-1.389698000	0.090712000
F	-4.641476000	-2.686533000	0.036126000
F	-4.772097000	-0.895728000	1.248272000
F	-4.939002000	-0.761635000	-0.911674000

MVK-Cat1-NO_2
E **=** -1252.662879
H **=** -1252.640294
G **=** -1252.719117
N_{imag} **=** 0
SP **=** -1253.9411741

C	-6.885461000	-0.106751000	-0.221325000
H	-7.105174000	-1.042629000	-0.744924000
C	-7.865123000	0.744853000	0.114467000
H	-8.908765000	0.525274000	-0.126541000

H	-7.671117000	1.685172000	0.637900000
C	-5.443001000	0.096927000	0.059527000
C	-4.991515000	1.344956000	0.780555000
H	-5.301311000	2.244067000	0.225237000
H	-5.459373000	1.400554000	1.776237000
H	-3.900297000	1.342351000	0.891330000
O	-4.648153000	-0.764644000	-0.304725000
C	0.263325000	-1.087219000	0.017314000
C	1.031869000	-1.216700000	-1.176943000
C	2.447426000	-1.276283000	-1.121624000
C	3.082551000	-1.198577000	0.145266000
C	2.342294000	-1.091127000	1.351695000
C	0.927271000	-1.030447000	1.278430000
H	3.048380000	-1.332196000	-2.028615000
H	2.864162000	-1.011266000	2.304688000
H	0.353198000	-0.892192000	2.195753000
I	-1.856018000	-0.926663000	-0.088599000
H	0.537920000	-1.221350000	-2.149577000
C	3.074253000	2.350549000	-0.235250000
C	2.287917000	2.466262000	0.955900000
C	2.181249000	2.296077000	-1.352438000
H	4.161648000	2.298708000	-0.282409000
C	0.907098000	2.485641000	0.573240000
H	2.672128000	2.524385000	1.973425000
C	0.841110000	2.380114000	-0.852452000
H	2.469841000	2.199742000	-2.398397000
H	0.055684000	2.556894000	1.249019000
H	-0.069170000	2.354518000	-1.450371000
Ru	1.788439000	0.568516000	-0.043592000
N	4.567660000	-1.158312000	0.205301000
O	5.076655000	-1.139896000	1.310061000
O	5.165082000	-1.142344000	-0.854616000

MVK-Cat1-CN
***E* =** -1140.492024
***H* =** -1140.470145
***G* =** -1140.547322
N_{imag} = 0
***SP* =** -114 1.6295104

C	-6.728877000	0.818123000	-0.000433000
H	-6.587332000	1.903611000	-0.000421000
C	-7.957210000	0.280486000	-0.000824000
H	-8.848649000	0.913588000	-0.001118000
H	-8.124379000	-0.800181000	-0.000808000
C	-5.459612000	0.049268000	-0.000002000
C	-5.501787000	-1.460186000	0.000019000
H	-6.042786000	-1.827976000	0.886059000
H	-6.041703000	-1.827961000	-0.886695000
H	-4.483191000	-1.866870000	0.000635000
O	-4.399966000	0.668971000	0.000278000
C	0.297069000	-0.863971000	0.000089000
C	0.985372000	-1.068788000	-1.230397000
C	2.344001000	-1.473414000	-1.238006000
C	3.032784000	-1.678422000	-0.000187000
C	2.344191000	-1.473663000	1.237767000
C	0.985574000	-1.069023000	1.230425000
H	2.874526000	-1.590960000	-2.183647000
H	2.874854000	-1.591490000	2.183296000
H	0.483887000	-0.871474000	2.178872000
I	-1.733436000	-0.230753000	0.000323000
H	0.483549000	-0.871055000	-2.178733000

C	3.844331000	1.819445000	-0.000036000
C	3.033739000	2.043241000	1.158630000
C	3.033785000	2.043053000	-1.158758000
H	4.891583000	1.518609000	0.000040000
C	1.720874000	2.407123000	0.715626000
H	3.357488000	1.949994000	2.194459000
C	1.720906000	2.406996000	-0.715857000
H	3.357602000	1.949595000	-2.194548000
H	0.867701000	2.633706000	1.353983000
H	0.867749000	2.633476000	-1.354272000
Ru	2.173250000	0.377781000	0.000015000
C	4.424269000	-2.042868000	-0.000368000
N	5.543069000	-2.346178000	-0.000463000

MVK-Cat1-Mn
***E* =** -2104.406758
***H* =** -2104.386848
***G* =** -2104.460108
N_{imag} = 0
***SP* =** -2105.5640678

C	-6.300722000	1.028275000	-0.019162000
H	-6.170182000	2.114732000	-0.059822000
C	-7.523811000	0.480386000	0.005036000
H	-8.421996000	1.103803000	-0.015307000
H	-7.679615000	-0.601307000	0.045592000
C	-5.018511000	0.274310000	0.004969000
C	-5.049355000	-1.236948000	0.061318000
H	-5.579398000	-1.576850000	0.965260000
H	-5.592387000	-1.643607000	-0.806539000
H	-4.025231000	-1.630004000	0.068644000
O	-3.966281000	0.898618000	-0.020956000
C	0.875819000	-0.971466000	-0.012159000
C	1.550707000	-1.177713000	-1.246437000
C	2.903768000	-1.612971000	-1.239973000
C	3.577026000	-1.836300000	-0.009955000
C	2.896205000	-1.631365000	1.219435000
C	1.542525000	-1.196509000	1.223585000
H	3.446777000	-1.691509000	-2.183579000
H	4.638844000	-2.087872000	-0.008755000
H	3.432819000	-1.726319000	2.165148000
H	1.044956000	-0.967273000	2.166820000
I	-1.129354000	-0.224206000	-0.012347000
H	1.060554000	-0.931815000	-2.189361000
C	4.350866000	1.367841000	-0.084345000
C	3.641362000	1.570980000	1.138603000
C	3.459461000	1.650971000	-1.163540000
H	5.386970000	1.044362000	-0.177175000
C	2.311828000	1.981045000	0.814549000
H	4.041326000	1.428807000	2.141856000
C	2.199389000	2.029561000	-0.607602000
H	3.695389000	1.579338000	-2.224693000
H	1.516230000	2.199961000	1.525747000
H	1.301654000	2.288404000	-1.167827000
Mn	2.679598000	0.058821000	0.002804000

MVK-Cat1-Tc
***E* =** -1034.383016
***H* =** -1034.362692
***G* =** -1034.438191
N_{imag} = 0
***SP* =** -1035.4127701

C	-6.536389000	1.141267000	0.007566000
H	-6.378905000	2.204950000	-0.199472000
C	-7.762963000	0.660079000	0.253796000
H	-8.637364000	1.316763000	0.253966000
H	-7.944911000	-0.397582000	0.464136000
C	-5.284092000	0.338458000	-0.012323000
C	-5.352902000	-1.148527000	0.256145000
H	-5.756944000	-1.336821000	1.263766000
H	-6.027327000	-1.641169000	-0.461925000
H	-4.350128000	-1.586679000	0.177592000
O	-4.223891000	0.904244000	-0.243066000
C	0.599045000	-1.045456000	-0.043383000
C	1.312295000	-1.302098000	-1.252998000
C	2.647875000	-1.797964000	-1.186516000
C	3.262341000	-2.034677000	0.077189000
C	2.540058000	-1.788284000	1.281159000
C	1.203089000	-1.293213000	1.226088000
H	3.212815000	-1.953741000	-2.108291000
H	4.300715000	-2.369340000	0.123542000
H	3.021231000	-1.938657000	2.250141000
H	0.660146000	-1.072356000	2.147069000
I	-1.409498000	-0.294680000	-0.134411000
H	0.853514000	-1.087447000	-2.220010000
C	4.258534000	1.485698000	0.003559000
C	3.507360000	1.717401000	1.199263000
C	3.413279000	1.778238000	-1.113145000
H	5.300634000	1.170622000	-0.046982000
C	2.197973000	2.155499000	0.820984000
H	3.877394000	1.611311000	2.218776000
C	2.139826000	2.192581000	-0.607489000
H	3.698197000	1.724685000	-2.163658000
H	1.392804000	2.433692000	1.500569000
H	1.281269000	2.500021000	-1.204185000
Tc	2.491149000	0.057019000	0.037533000

MVK-Cat1-Re
***E* =** -1031.851534
***H* =** -1031.831056
***G* =** -1031.907177
N_{imag} = 0
***SP* =** -1032.8727535

C	-6.877214000	1.160223000	-0.023528000
H	-6.723914000	2.195576000	-0.345393000
C	-8.095116000	0.716447000	0.317661000
H	-8.966430000	1.376323000	0.283160000
H	-8.272574000	-0.312317000	0.643437000
C	-5.629749000	0.349870000	-0.007807000
C	-5.694927000	-1.101886000	0.411818000
H	-6.071951000	-1.186544000	1.443570000
H	-6.389807000	-1.659950000	-0.235476000
H	-4.695855000	-1.551072000	0.350240000
O	-4.576593000	0.880571000	-0.334165000
C	0.255270000	-1.057441000	-0.070261000
C	0.981723000	-1.313337000	-1.274483000
C	2.313410000	-1.824159000	-1.196494000
C	2.911577000	-2.076003000	0.074165000
C	2.177609000	-1.828996000	1.273317000
C	0.844189000	-1.319272000	1.205781000
H	2.887643000	-1.978061000	-2.112297000
H	3.945746000	-2.420517000	0.130029000
H	2.646181000	-1.990582000	2.246165000

H	0.292376000	-1.100165000	2.121530000
I	-1.760053000	-0.316853000	-0.181067000
H	0.536261000	-1.087891000	-2.244919000
C	3.968575000	1.473181000	0.005541000
C	3.232256000	1.703586000	1.212495000
C	3.116617000	1.792616000	-1.100543000
H	5.005415000	1.144974000	-0.058718000
C	1.925428000	2.167938000	0.851603000
H	3.611817000	1.583990000	2.226561000
C	1.854043000	2.222265000	-0.577084000
H	3.391487000	1.749755000	-2.153799000
H	1.133583000	2.456055000	1.542103000
H	0.996667000	2.554495000	-1.161422000
Re	2.171557000	0.047792000	0.039760000

MVK-Cat1-Fe
E **=** -2216.970069
H **=** -2216.950365
G **=** -2217.023524
$\boldsymbol{N_{imag}}$ **=** 0
SP **=** -2218.1445372

C	-6.150337000	1.065706000	-0.018837000
H	-5.963745000	2.141342000	-0.100326000
C	-7.399509000	0.583887000	0.051958000
H	-8.263469000	1.253687000	0.029998000
H	-7.611350000	-0.485638000	0.134570000
C	-4.913895000	0.244614000	0.004765000
C	-5.017902000	-1.258241000	0.114408000
H	-5.532962000	-1.538198000	1.047031000
H	-5.610933000	-1.665535000	-0.719452000
H	-4.016775000	-1.706360000	0.102933000
O	-3.830072000	0.815581000	-0.063978000
C	0.814745000	-0.961129000	-0.023699000
C	1.515426000	-1.160543000	-1.241874000
C	2.862545000	-1.598329000	-1.226731000
C	3.522882000	-1.831079000	0.002489000
C	2.831265000	-1.622856000	1.218910000
C	1.483782000	-1.185527000	1.207927000
H	3.412549000	-1.671597000	-2.165490000
H	4.582673000	-2.086145000	0.013225000
H	3.356661000	-1.716501000	2.169808000
H	0.987188000	-0.951675000	2.149916000
I	-1.184051000	-0.228856000	-0.039703000
H	1.043925000	-0.905805000	-2.191272000
C	4.317306000	1.323935000	-0.070378000
C	3.612301000	1.527218000	1.154635000
C	3.427992000	1.624335000	-1.146164000
H	5.339570000	0.962522000	-0.167529000
C	2.287290000	1.953461000	0.835343000
H	4.003189000	1.347578000	2.154697000
C	2.173565000	2.013034000	-0.585884000
H	3.652766000	1.532388000	-2.207428000
H	1.488142000	2.151107000	1.548032000
H	1.272230000	2.262765000	-1.143525000
Fe	2.655804000	0.091149000	0.011525000

MVK-Cat1-Os
E **=** -1044.109948
H **=** -1044.089857
G **=** -1044.163347
$\boldsymbol{N_{imag}}$ **=** 0

***SP* =** -1045.1358115

C	-6.722060000	1.139847000	-0.149658000
H	-6.530944000	2.111492000	-0.616388000
C	-7.962218000	0.775968000	0.206917000
H	-8.814022000	1.440796000	0.039682000
H	-8.178411000	-0.187935000	0.675787000
C	-5.501464000	0.315441000	0.035325000
C	-5.614888000	-1.055368000	0.659711000
H	-6.033766000	-0.979117000	1.675697000
H	-6.297814000	-1.690034000	0.073372000
H	-4.627296000	-1.530158000	0.708010000
O	-4.423248000	0.775849000	-0.326469000
C	0.193174000	-1.063565000	-0.105655000
C	0.934770000	-1.267713000	-1.307206000
C	2.264653000	-1.774929000	-1.251676000
C	2.865596000	-2.071210000	0.002591000
C	2.134382000	-1.857530000	1.203608000
C	0.803898000	-1.350680000	1.151256000
H	2.838404000	-1.883121000	-2.172880000
H	3.902186000	-2.406634000	0.046074000
H	2.606578000	-2.032111000	2.171171000
H	0.267905000	-1.144652000	2.078349000
I	-1.799833000	-0.307144000	-0.185216000
H	0.500492000	-0.997374000	-2.270260000
C	3.930434000	1.405105000	0.028576000
C	3.210330000	1.604481000	1.252733000
C	3.069180000	1.774360000	-1.057034000
H	4.953168000	1.042278000	-0.060463000
C	1.904466000	2.097144000	0.922981000
H	3.590380000	1.420405000	2.256366000
C	1.817047000	2.201383000	-0.503318000
H	3.322852000	1.741363000	-2.115391000
H	1.115689000	2.346710000	1.630934000
H	0.949163000	2.540724000	-1.066862000
Os	2.148010000	0.066352000	0.037370000

MVK-Cat1-Co

***E* =** -2335.785469

***H* =** -2335.766042

***G* =** -2335.835706

N_{imag} = 0

***SP* =** -2336.9803891

C	6.035604000	1.107942000	-0.125929000
H	5.796977000	2.133118000	-0.425825000
C	7.304799000	0.721640000	0.070861000
H	8.132337000	1.423225000	-0.064249000
H	7.569772000	-0.296046000	0.370249000
C	4.848107000	0.236982000	0.029395000
C	5.019481000	-1.204595000	0.437610000
H	5.639875000	-1.738405000	-0.299366000
H	5.538378000	-1.268910000	1.406740000
H	4.042801000	-1.697804000	0.514740000
O	3.738463000	0.725194000	-0.183748000
C	-0.765072000	-0.964471000	-0.079360000
C	-1.439026000	-1.232308000	1.146092000
C	-2.784694000	-1.673119000	1.155259000
C	-3.492519000	-1.828139000	-0.060387000
C	-2.844463000	-1.537679000	-1.284533000
C	-1.498355000	-1.098655000	-1.292474000
H	-3.298304000	-1.809972000	2.107908000
H	-4.551650000	-2.087559000	-0.047488000

H	-3.405802000	-1.566242000	-2.219675000
H	-1.042712000	-0.797782000	-2.237105000
I	1.241476000	-0.273956000	-0.096356000
H	-0.936285000	-1.040423000	2.095118000
C	-4.282966000	1.354986000	-0.132142000
C	-3.277585000	1.734213000	-1.074888000
C	-3.716444000	1.443406000	1.177030000
H	-5.283568000	0.998187000	-0.373324000
C	-2.089149000	2.058401000	-0.347862000
H	-3.379790000	1.719630000	-2.159378000
C	-2.361007000	1.881273000	1.043434000
H	-4.212562000	1.171431000	2.108242000
H	-1.125337000	2.324857000	-0.781727000
H	-1.642500000	1.998363000	1.854453000
Co	-2.647696000	0.120488000	0.027959000

MVK-Cat1-Rh
***E* =** -1063.741023
***H* =** -1063.721039
***G* =** -1063.794816
N_{imag} = 0
***SP* =** -1064.7842394

C	-6.266666000	1.205561000	-0.134050000
H	-6.014880000	2.192279000	-0.535139000
C	-7.533194000	0.882632000	0.166532000
H	-8.345875000	1.598591000	0.017329000
H	-7.810538000	-0.095755000	0.568447000
C	-5.098630000	0.309869000	0.032413000
C	-5.293190000	-1.085185000	0.571724000
H	-5.759759000	-1.049000000	1.568557000
H	-5.970390000	-1.655579000	-0.083294000
H	-4.330470000	-1.606347000	0.639175000
O	-3.988070000	0.738238000	-0.279252000
C	0.479138000	-1.081974000	-0.086153000
C	1.228703000	-1.281328000	-1.283949000
C	2.558175000	-1.775668000	-1.234608000
C	3.172156000	-2.053083000	0.013878000
C	2.444274000	-1.840532000	1.213060000
C	1.112713000	-1.351571000	1.163521000
H	3.124849000	-1.890344000	-2.160656000
H	4.210828000	-2.385104000	0.053310000
H	2.922169000	-2.006560000	2.180356000
H	0.582770000	-1.149089000	2.096491000
I	-1.505623000	-0.337235000	-0.156571000
H	0.789732000	-1.024637000	-2.249974000
C	4.206898000	1.439927000	0.046134000
C	3.458681000	1.643471000	1.251198000
C	3.363875000	1.779765000	-1.061942000
H	5.228471000	1.065284000	-0.017648000
C	2.153391000	2.114123000	0.887572000
H	3.812671000	1.456546000	2.264921000
C	2.093937000	2.196223000	-0.541066000
H	3.632719000	1.712147000	-2.116127000
H	1.338045000	2.340129000	1.574935000
H	1.223972000	2.492961000	-1.127596000
Rh	2.457483000	0.119081000	0.040747000

MVK-Cat1-CO
***E* =** -1194.516888
***H* =** -1194.494394
***G* =** -1194.572490

N

N_{imag} = 0

***SP* =** -1195.6802711

C	-6.474238000	0.180896000	0.035570000
H	-6.763578000	-0.874450000	0.046127000
C	-7.397281000	1.153677000	0.066240000
H	-8.463429000	0.914536000	0.102621000
H	-7.133596000	2.214756000	0.056134000
C	-5.011911000	0.402018000	-0.011744000
C	-4.463388000	1.806638000	-0.026927000
H	-4.853364000	2.358024000	-0.896639000
H	-4.785832000	2.350939000	0.874529000
H	-3.367538000	1.793419000	-0.069207000
O	-4.276743000	-0.586230000	-0.036511000
C	0.442974000	-1.227778000	0.023582000
C	1.191446000	-1.388796000	-1.182778000
C	2.562348000	-1.751728000	-1.139655000
C	3.238557000	-1.882377000	0.100168000
C	2.515748000	-1.666078000	1.302076000
C	1.147162000	-1.297459000	1.266300000
H	3.110245000	-1.889249000	-2.075147000
H	4.297749000	-2.141964000	0.129113000
H	3.025311000	-1.741580000	2.266003000
H	0.621054000	-1.109693000	2.204147000
I	-1.641285000	-0.871756000	-0.023637000
H	0.701051000	-1.271415000	-2.150820000
Ru	2.360608000	0.289031000	0.005565000
C	3.397817000	1.223270000	1.351410000
C	3.417964000	1.108284000	-1.395506000
C	1.097912000	1.763480000	-0.048681000
O	0.326203000	2.588583000	-0.075274000
O	4.038107000	1.550384000	-2.230168000
O	4.005263000	1.739523000	2.152042000

MVK-Cat1-NCH

***E* =** -1134.860673

***H* =** -1134.836686

***G* =** -1134.916190

N_{imag} = 0

***SP* =** -1135.9795602

C	6.450013000	0.229260000	0.127609000
H	6.761402000	-0.792285000	0.367416000
C	7.352622000	1.206310000	-0.042811000
H	8.422998000	1.005766000	0.054039000
H	7.067145000	2.233881000	-0.284060000
C	4.981929000	0.396525000	0.014735000
C	4.405355000	1.754641000	-0.308258000
H	4.714067000	2.491728000	0.449479000
H	4.784249000	2.108898000	-1.279960000
H	3.309858000	1.704816000	-0.342516000
O	4.267091000	-0.588109000	0.188245000
C	-0.531171000	-1.279102000	-0.094650000
C	-1.298288000	-1.502060000	1.085396000
C	-2.695171000	-1.751264000	0.984340000
C	-3.352287000	-1.744371000	-0.272236000
C	-2.587023000	-1.476772000	-1.438848000
C	-1.192165000	-1.225275000	-1.359151000
H	-3.279285000	-1.877538000	1.898838000
H	-4.430007000	-1.896522000	-0.336946000
H	-3.087773000	-1.389736000	-2.405780000
H	-0.633371000	-0.987443000	-2.265327000
I	1.554908000	-0.936350000	0.029839000

H	-0.822771000	-1.477684000	2.066696000
Ru	-2.279356000	0.211231000	-0.003775000
N	-3.277325000	1.439041000	-1.287913000
N	-3.321248000	1.133592000	1.483148000
N	-0.927876000	1.726911000	0.181241000
C	-0.137147000	2.554780000	0.285222000
C	-3.913241000	1.645447000	2.325158000
C	-3.841295000	2.124338000	-2.018488000
H	0.615216000	3.329635000	0.382734000
H	-4.472005000	2.124436000	3.121792000
H	-4.372556000	2.767780000	-2.711201000

MVK-Cat1-CNH
***E* =** -1134.884479
***H* =** -1134.860715
***G* =** -1134.939212
N_{imag} = 0
***SP* =** -1136.0003328

C	6.426771000	0.319649000	0.192399000
H	6.762532000	-0.666258000	0.528918000
C	7.301813000	1.312569000	-0.023923000
H	8.373386000	1.160924000	0.131101000
H	6.991855000	2.305130000	-0.362039000
C	4.959862000	0.421362000	0.008922000
C	4.350913000	1.725010000	-0.450926000
H	4.620121000	2.539786000	0.239036000
H	4.743885000	1.996799000	-1.443437000
H	3.258369000	1.639297000	-0.505473000
O	4.273569000	-0.572691000	0.236774000
C	-0.511737000	-1.311078000	-0.107277000
C	-1.285283000	-1.574373000	1.047102000
C	-2.685358000	-1.830052000	0.930099000
C	-3.319792000	-1.800873000	-0.325998000
C	-2.545633000	-1.504228000	-1.487387000
C	-1.165303000	-1.245853000	-1.383293000
H	-3.269500000	-2.023105000	1.832178000
H	-4.391590000	-1.983968000	-0.409597000
H	-3.029609000	-1.445629000	-2.464282000
H	-0.592504000	-1.003715000	-2.279838000
I	1.579305000	-0.983904000	0.036141000
H	-0.817489000	-1.590923000	2.032590000
Ru	-2.295215000	0.285979000	0.004605000
C	-3.551342000	1.354064000	-1.032334000
C	-2.991027000	1.138026000	1.614355000
C	-0.995845000	1.732202000	-0.145021000
N	-0.179304000	2.551986000	-0.238002000
N	-3.409283000	1.623101000	2.582113000
N	-4.308311000	1.968030000	-1.662633000
H	0.543835000	3.254478000	-0.313891000
H	-3.774966000	2.044789000	3.425221000
H	-4.970551000	2.497404000	-2.213406000

TS-1
***E* =** -464.103555
***H* =** -464.092111
***G* =** -464.139309
N_{imag} = 1, -457 cm^{-1}
***SP* =**-464.8388129

C	-0.530441000	1.070408000	0.938834000
C	-1.663806000	1.481898000	0.031497000
C	-2.382330000	0.272367000	-0.631073000

C	-1.732465000	-1.058941000	-0.290389000
C	-1.206902000	-1.233115000	0.995659000
C	-0.565970000	-0.140089000	1.609523000
H	-3.423769000	0.223463000	-0.271912000
H	-2.383782000	2.036641000	0.659434000
H	-1.312764000	2.206834000	-0.717529000
H	0.137207000	1.863012000	1.287497000
H	-2.159396000	-1.931786000	-0.794778000
H	-1.074300000	-2.239482000	1.403687000
H	0.073473000	-0.303388000	2.481415000
H	-2.445699000	0.398444000	-1.722765000
C	0.754735000	0.264006000	-1.013401000
H	0.567715000	1.225610000	-1.495987000
C	0.032038000	-0.865894000	-1.419567000
H	-0.546230000	-0.796019000	-2.343969000
H	0.438781000	-1.859408000	-1.223625000
C	2.004023000	0.231049000	-0.240575000
C	2.436089000	-1.092016000	0.371150000
H	1.604820000	-1.603495000	0.878870000
H	2.795555000	-1.767456000	-0.423438000
H	3.254673000	-0.911485000	1.080383000
O	2.700487000	1.235058000	-0.113404000

TS-1 (exo)
***E* =** -464.099394
***H* =** -464.087899
***G* =** -464.135229
N_{imag} = 1, -470 cm^{-1}
***SP* =** -464.83455

C	-0.644130000	1.280584000	0.688184000
C	-1.728789000	1.423419000	-0.159935000
C	-2.351641000	0.263197000	-0.656373000
C	-1.792765000	-0.980669000	-0.337009000
C	-1.216445000	-1.182969000	1.053447000
C	-0.568713000	0.106897000	1.633349000
H	-1.948697000	2.393964000	-0.614589000
H	-0.007021000	2.139909000	0.913540000
H	-2.222202000	-1.874804000	-0.800299000
H	-0.508713000	-2.025974000	1.052785000
H	0.468187000	-0.063128000	1.957372000
H	-3.073046000	0.344478000	-1.474899000
C	0.754426000	0.249840000	-1.057769000
H	0.653265000	1.189261000	-1.603494000
C	-0.003941000	-0.860405000	-1.460841000
H	-0.503272000	-0.816030000	-2.429780000
H	0.324730000	-1.862998000	-1.175496000
C	1.970589000	0.206336000	-0.231745000
C	2.444786000	-1.125994000	0.330931000
H	1.630407000	-1.733029000	0.749878000
H	2.903258000	-1.717226000	-0.479414000
H	3.202932000	-0.938432000	1.102593000
O	2.632080000	1.221741000	-0.033366000
H	-1.108910000	0.405458000	2.549316000
H	-2.047820000	-1.496784000	1.705986000

TS-cat1
***E* =** -1281.446384
***H* =** -1281.421526
***G* =** -1281.505822
N_{imag} = 1, -444 cm^{-1}
***SP* =** -1282.8570722

C	-4.894275000	1.367564000	0.872992000
C	-5.879008000	2.290121000	0.201593000
C	-7.279251000	1.646736000	-0.002136000
C	-7.329980000	0.186089000	0.418102000
C	-6.580799000	-0.209373000	1.535223000
C	-5.316688000	0.377065000	1.739374000
H	-8.021208000	2.180936000	0.613676000
H	-5.972637000	3.176548000	0.854705000
H	-5.463074000	2.670701000	-0.742666000
H	-3.845885000	1.678477000	0.875077000
H	-8.287781000	-0.315155000	0.245414000
H	-6.855931000	-1.112363000	2.087839000
H	-4.608183000	-0.086006000	2.431434000
H	-7.616417000	1.755767000	-1.044207000
C	-4.975106000	-0.099431000	-1.212834000
H	-4.840460000	0.791398000	-1.829321000
C	-6.247609000	-0.683498000	-1.109660000
H	-6.997024000	-0.379624000	-1.844911000
H	-6.331331000	-1.727719000	-0.803042000
C	-3.726235000	-0.718510000	-0.797716000
C	-3.767683000	-2.015756000	-0.016175000
H	-4.595501000	-2.045884000	0.705529000
H	-3.915941000	-2.851776000	-0.720729000
H	-2.815302000	-2.169419000	0.507857000
O	-2.641418000	-0.211366000	-1.123089000
C	1.951463000	-1.129699000	0.228872000
C	2.893471000	-1.621340000	-0.720676000
C	4.251118000	-1.820255000	-0.348189000
C	4.680582000	-1.523185000	0.972419000
C	3.746703000	-1.029402000	1.921797000
C	2.388183000	-0.829880000	1.551540000
H	4.972108000	-2.155441000	-1.095734000
H	5.732003000	-1.629229000	1.243570000
H	4.077872000	-0.757108000	2.925390000
H	1.689771000	-0.406143000	2.274883000
I	-0.079001000	-0.783012000	-0.342103000
H	2.584238000	-1.806964000	-1.750501000
C	5.208696000	1.860931000	-0.148546000
C	4.200629000	2.338058000	0.749432000
C	4.586638000	1.592374000	-1.410386000
H	6.263488000	1.725887000	0.087417000
C	2.954977000	2.366513000	0.041531000
H	4.353604000	2.629237000	1.787804000
C	3.193482000	1.905495000	-1.292589000
H	5.084858000	1.215599000	-2.302718000
H	1.993063000	2.675693000	0.448591000
H	2.443262000	1.799658000	-2.075313000
Ru	3.680340000	0.291587000	0.118007000

TS-cat1 (exo)
***E* =** -1281.441843
***H* =** -1281.417112
***G* =** -1281.499853
***N_{imag}* =** 1, -448 cm^{-1}
***SP* =** -1282.852201

C	-5.028457000	1.777810000	-0.067203000
C	-6.211414000	2.056788000	-0.727941000
C	-7.375253000	1.350587000	-0.359787000
C	-7.259025000	0.320056000	0.587917000
C	-6.299168000	0.508841000	1.752081000
C	-5.037366000	1.327071000	1.368631000

H	-6.202158000	2.641961000	-1.652460000
H	-4.077197000	2.131236000	-0.473966000
H	-8.161848000	-0.258312000	0.818594000
H	-6.030709000	-0.464997000	2.183967000
H	-4.109795000	0.787474000	1.611492000
H	-8.272629000	1.426480000	-0.979649000
C	-5.028410000	-0.606015000	-1.010620000
H	-4.983158000	-0.139379000	-1.996051000
C	-6.266709000	-1.093637000	-0.548539000
H	-7.059540000	-1.233511000	-1.291118000
H	-6.275135000	-1.844443000	0.246438000
C	-3.717993000	-0.914042000	-0.471841000
C	-3.554100000	-1.746042000	0.787274000
H	-4.476917000	-1.911729000	1.350364000
H	-3.139625000	-2.725949000	0.496637000
H	-2.809304000	-1.264544000	1.441470000
O	-2.696706000	-0.523407000	-1.066063000
C	1.999483000	-1.187691000	0.121183000
C	2.946898000	-1.527458000	-0.894666000
C	4.322191000	-1.671760000	-0.569479000
C	4.767463000	-1.474410000	0.764696000
C	3.828466000	-1.130369000	1.774490000
C	2.452720000	-0.982711000	1.457672000
H	5.042855000	-1.892719000	-1.357498000
H	5.828766000	-1.537693000	1.005605000
H	4.172461000	-0.931945000	2.791279000
H	1.747710000	-0.676586000	2.228661000
I	-0.061863000	-0.927297000	-0.372433000
H	2.619735000	-1.634853000	-1.926425000
C	5.032981000	2.053890000	0.109235000
C	3.904072000	2.369491000	0.934678000
C	4.562247000	1.835776000	-1.224572000
H	6.069529000	1.987285000	0.443786000
C	2.734224000	2.351029000	0.101310000
H	3.930049000	2.591503000	2.002712000
C	3.141340000	2.022981000	-1.227723000
H	5.177175000	1.577261000	-2.086385000
H	1.709163000	2.543097000	0.429699000
H	2.484126000	1.922128000	-2.093436000
Ru	3.637137000	0.343451000	0.104691000
H	-4.994646000	2.245934000	1.984315000
H	-6.855651000	1.043340000	2.542250000

TS-cat2

***E* =** -993.304526

***H* =** -993.285086

***G* =** -993.356024

N_{imag} = 1, -452 cm^{-1}

***SP* =** -994.3916828

C	-2.170185000	1.042681000	-0.770549000
C	-3.310214000	1.552506000	-1.614920000
C	-4.590190000	1.859592000	-0.788264000
C	-4.455887000	1.467399000	0.674453000
C	-3.216010000	1.660835000	1.299978000
C	-2.046282000	1.412241000	0.557277000
H	-4.792066000	2.943315000	-0.813259000
H	-2.953292000	2.480158000	-2.097437000
H	-3.514856000	0.856924000	-2.441970000
H	-1.304130000	0.632360000	-1.296464000
H	-5.359831000	1.597752000	1.278235000
H	-3.158837000	1.757569000	2.388200000

H	-1.085873000	1.293649000	1.067073000
H	-5.473688000	1.377098000	-1.233350000
C	-3.443090000	-1.054523000	-0.330461000
H	-3.584252000	-1.141951000	-1.409498000
C	-4.500256000	-0.609731000	0.478220000
H	-5.497744000	-0.573491000	0.033318000
H	-4.499037000	-0.860084000	1.540482000
C	-2.232348000	-1.713604000	0.161334000
C	-2.008803000	-1.784922000	1.660895000
H	-2.151711000	-0.805603000	2.141857000
H	-2.740607000	-2.475829000	2.111949000
H	-0.995920000	-2.155007000	1.864317000
O	-1.420086000	-2.218470000	-0.618405000
C	3.143653000	0.189565000	-0.003475000
C	4.387307000	-0.438844000	-0.141616000
C	5.557588000	0.313536000	0.012927000
C	5.488361000	1.680322000	0.302124000
C	4.240753000	2.298274000	0.437597000
C	3.062947000	1.557609000	0.285700000
H	6.528339000	-0.177280000	-0.095124000
H	6.405001000	2.262899000	0.421561000
H	4.177357000	3.365942000	0.663364000
H	2.091986000	2.046062000	0.391846000
I	1.346737000	-0.939041000	-0.236358000
H	4.448690000	-1.505184000	-0.368181000

TS-Cat1-CF_3
***E* =** -1618.229672
***H* =** -1618.201185
***G* =** -1618.293678
N_{imag} = 1, -433 cm^{-1}
***SP* =** -1620.0479845

C	-5.979731000	-0.778533000	1.356860000
C	-7.080706000	0.031924000	1.990714000
C	-8.347120000	0.149389000	1.096583000
C	-8.172756000	-0.495473000	-0.269467000
C	-7.405695000	-1.665659000	-0.359441000
C	-6.262347000	-1.785700000	0.453800000
H	-9.191202000	-0.362897000	1.586647000
H	-7.342054000	-0.482406000	2.933145000
H	-6.702659000	1.018555000	2.295736000
H	-4.996655000	-0.739329000	1.834057000
H	-9.027757000	-0.393992000	-0.945473000
H	-7.547192000	-2.349740000	-1.201172000
H	-5.510641000	-2.546092000	0.225771000
H	-8.655427000	1.200012000	0.984429000
C	-5.744503000	1.050523000	-0.398838000
H	-5.734913000	1.798008000	0.396790000
C	-6.906447000	0.872393000	-1.166508000
H	-7.668197000	1.653821000	-1.106207000
H	-6.829532000	0.408291000	-2.151407000
C	-4.429444000	0.524012000	-0.725180000
C	-4.285075000	-0.459798000	-1.868164000
H	-5.155192000	-1.121068000	-1.973882000
H	-4.181679000	0.106030000	-2.809825000
H	-3.379394000	-1.064689000	-1.729627000
O	-3.429569000	0.914255000	-0.100254000
C	1.178710000	-0.603070000	-0.093447000
C	1.934429000	-0.753483000	-1.291998000
C	3.293604000	-1.158186000	-1.242344000
C	3.911412000	-1.404550000	0.016547000

C	3.159021000	-1.280725000	1.218512000
C	1.801382000	-0.872287000	1.159636000
H	3.878459000	-1.216796000	-2.161384000
H	3.641853000	-1.437271000	2.183590000
H	1.249458000	-0.720028000	2.088417000
I	-0.853722000	0.051611000	-0.162450000
H	1.486162000	-0.507356000	-2.255815000
C	4.751298000	2.038742000	0.045939000
C	4.046504000	2.173791000	1.285749000
C	3.849711000	2.374244000	-1.014145000
H	5.787407000	1.723590000	-0.071637000
C	2.709051000	2.594512000	0.990405000
H	4.455157000	1.986904000	2.278002000
C	2.587250000	2.716917000	-0.429922000
H	4.081387000	2.364075000	-2.078445000
H	1.919205000	2.778246000	1.717665000
H	1.687487000	3.006166000	-0.971707000
Ru	3.063342000	0.625037000	0.068676000
C	5.376467000	-1.784829000	0.072580000
F	5.530033000	-3.101274000	-0.126144000
F	6.081073000	-1.145129000	-0.874608000
F	5.913629000	-1.482265000	1.262445000

TS-Cat1-NO_2
***E* =** -1485.783556
***H* =** -1485.756110
***G* =** -1485.846162
N_{imag} = 1, -437 cm^{-1}
***SP* =** -1487.4384099

C	-5.753641000	0.770785000	1.356179000
C	-6.816174000	1.770412000	0.979787000
C	-8.095270000	1.113330000	0.389915000
C	-7.969494000	-0.393284000	0.227123000
C	-7.240978000	-1.107857000	1.188766000
C	-6.084680000	-0.516640000	1.733941000
H	-8.945802000	1.291739000	1.067938000
H	-7.075253000	2.312185000	1.907425000
H	-6.401753000	2.534792000	0.306320000
H	-4.760237000	1.164753000	1.587721000
H	-8.836268000	-0.886925000	-0.223855000
H	-7.421104000	-2.177592000	1.328956000
H	-5.361023000	-1.134339000	2.272476000
H	-8.370570000	1.577602000	-0.569190000
C	-5.504029000	0.221952000	-1.125549000
H	-5.457055000	1.290136000	-1.345946000
C	-6.687215000	-0.488025000	-1.387401000
H	-7.424953000	-0.004643000	-2.032898000
H	-6.642576000	-1.572151000	-1.506143000
C	-4.212375000	-0.374438000	-0.831227000
C	-4.121154000	-1.866965000	-0.589000000
H	-5.010321000	-2.270469000	-0.086636000
H	-4.028067000	-2.379434000	-1.561744000
H	-3.227952000	-2.096350000	0.006705000
O	-3.188138000	0.329303000	-0.824679000
C	1.454946000	-0.818180000	-0.086198000
C	2.304105000	-1.084230000	-1.200455000
C	3.676588000	-1.385140000	-1.010610000
C	4.190178000	-1.408052000	0.312302000
C	3.368091000	-1.157918000	1.442082000
C	1.998238000	-0.857088000	1.231880000
H	4.344095000	-1.552954000	-1.855007000

H	3.803581000	-1.156712000	2.440637000
H	1.371229000	-0.610401000	2.089899000
I	-0.604039000	-0.339766000	-0.395395000
H	1.912423000	-1.013641000	-2.216249000
C	4.816816000	2.104484000	-0.046566000
C	3.956925000	2.350985000	1.071612000
C	4.030312000	2.192409000	-1.238966000
H	5.881310000	1.876320000	0.001916000
C	2.637599000	2.595496000	0.568357000
H	4.253450000	2.350143000	2.119713000
C	2.683200000	2.497373000	-0.858703000
H	4.391491000	2.046891000	-2.256219000
H	1.751654000	2.807632000	1.165580000
H	1.837644000	2.619366000	-1.534646000
Ru	3.242315000	0.557213000	0.012806000
N	5.643531000	-1.640933000	0.517271000
O	6.324984000	-1.802532000	-0.477887000
O	6.047108000	-1.651227000	1.665222000

TS-Cat1-CN
***E* =** -1373.612352
***H* =** -1373.585889
***G* =** -1373.671254
N_{imag} = 1, -449 cm^{-1}
***SP* =** -1375.1270858

C	-2.912262000	1.191039000	-0.379833000
C	-3.717903000	2.183210000	-1.176823000
C	-4.951517000	2.726926000	-0.403895000
C	-5.161403000	2.035381000	0.934153000
C	-4.025404000	1.697865000	1.686793000
C	-2.885983000	1.233264000	1.002708000
H	-4.808472000	3.798833000	-0.190542000
H	-3.036184000	3.018777000	-1.417887000
H	-4.000250000	1.752645000	-2.148647000
H	-2.142500000	0.630718000	-0.916450000
H	-6.059082000	2.342540000	1.480276000
H	-4.102988000	1.563459000	2.769423000
H	-2.098458000	0.707215000	1.550202000
H	-5.862876000	2.658096000	-1.016591000
C	-4.839052000	-0.437411000	-0.512469000
H	-4.842451000	-0.272600000	-1.591407000
C	-5.805869000	0.184724000	0.296141000
H	-6.671719000	0.623070000	-0.206472000
H	-6.037346000	-0.246564000	1.271635000
C	-3.961706000	-1.516616000	-0.078877000
C	-3.977186000	-1.925393000	1.380500000
H	-3.899402000	-1.056857000	2.051665000
H	-4.932910000	-2.424705000	1.611533000
H	-3.155445000	-2.624833000	1.580586000
O	-3.227308000	-2.103537000	-0.887985000
C	1.533628000	-1.465497000	0.056966000
C	2.491964000	-1.443794000	-0.996999000
C	3.818434000	-1.004177000	-0.755990000
C	4.200987000	-0.576942000	0.555347000
C	3.242213000	-0.603836000	1.617553000
C	1.919791000	-1.045461000	1.362428000
H	4.533891000	-0.951967000	-1.577741000
H	3.516825000	-0.245882000	2.610547000
H	1.185168000	-1.016104000	2.168271000
I	-0.489255000	-2.001065000	-0.329389000
H	2.199066000	-1.719835000	-2.011133000

C	3.043166000	2.697789000	-0.354203000
C	2.042237000	2.640143000	0.668302000
C	2.462063000	2.218149000	-1.571147000
H	4.071985000	3.032230000	-0.225220000
C	0.839646000	2.128808000	0.081045000
H	2.172614000	2.934081000	1.708926000
C	1.099576000	1.866612000	-1.301583000
H	2.967680000	2.133282000	-2.532229000
H	-0.107419000	1.959658000	0.592032000
H	0.386840000	1.461634000	-2.019326000
Ru	2.417080000	0.600002000	-0.076544000
C	5.529269000	-0.079434000	0.796060000
N	6.601823000	0.313664000	0.992988000

TS-Cat1-Mn

***E* =** -2337.527735

***H* =** -2337.503351

***G* =** -2337.583268

N_{imag} **=** 1, -449 cm^{-1}

***SP* =** -2339.0627319

C	-2.276725000	1.132203000	-0.459920000
C	-2.995810000	2.271019000	-1.135918000
C	-4.133249000	2.874630000	-0.264607000
C	-4.363963000	2.098777000	1.022084000
C	-3.245673000	1.583429000	1.690795000
C	-2.188117000	1.065636000	0.919819000
H	-3.866023000	3.904430000	0.025446000
H	-2.234380000	3.045779000	-1.337424000
H	-3.360813000	1.959615000	-2.125621000
H	-1.583333000	0.547462000	-1.069089000
H	-5.214095000	2.433631000	1.625249000
H	-3.296826000	1.363407000	2.761173000
H	-1.430545000	0.427741000	1.382661000
H	-5.070023000	2.954820000	-0.836892000
C	-4.308298000	-0.281211000	-0.648305000
H	-4.320951000	-0.036181000	-1.712221000
C	-5.206014000	0.353428000	0.223234000
H	-6.027755000	0.921894000	-0.219050000
H	-5.459428000	-0.126908000	1.169737000
C	-3.558683000	-1.501422000	-0.333591000
C	-3.589477000	-2.016236000	1.094331000
H	-3.374778000	-1.217612000	1.820331000
H	-4.595096000	-2.403860000	1.328384000
H	-2.859014000	-2.827609000	1.206417000
O	-2.933398000	-2.109021000	-1.204470000
C	1.976422000	-1.211216000	0.396076000
C	3.138069000	-1.342065000	-0.411991000
C	4.353443000	-0.740573000	0.013455000
C	4.403562000	-0.012000000	1.230381000
C	3.238340000	0.116470000	2.031380000
C	2.016493000	-0.481463000	1.616506000
H	5.224595000	-0.768283000	-0.643737000
H	5.314495000	0.521688000	1.506402000
H	3.254404000	0.742481000	2.925275000
H	1.105740000	-0.322068000	2.194957000
I	0.111039000	-2.011781000	-0.277171000
H	3.081503000	-1.834728000	-1.383370000
C	3.521353000	2.488824000	-0.758072000
C	2.522536000	2.763684000	0.225397000
C	2.921339000	1.702568000	-1.787901000
H	4.561085000	2.812169000	-0.722755000

C	1.304363000	2.149814000	-0.198482000
H	2.666914000	3.333720000	1.142327000
C	1.552054000	1.492865000	-1.441331000
H	3.422732000	1.319643000	-2.675975000
H	0.355771000	2.160397000	0.335953000
H	0.827295000	0.912981000	-2.011584000
Mn	2.798754000	0.665160000	0.059968000

TS-Cat1-Tc

***E* =** -1267.500988

***H* =** -1267.475979

***G* =** -1267.559956

N_{imag} = 1, -447 cm^{-1}

***SP* =** -1268.9074345

C	-5.093312000	1.303500000	0.925460000
C	-6.161405000	2.220226000	0.384014000
C	-7.524694000	1.505470000	0.164888000
C	-7.469447000	0.016678000	0.469156000
C	-6.657401000	-0.417608000	1.525045000
C	-5.423519000	0.228410000	1.731362000
H	-8.280209000	1.943027000	0.838180000
H	-6.286111000	3.029072000	1.126304000
H	-5.811099000	2.718672000	-0.531554000
H	-4.066023000	1.677637000	0.915825000
H	-8.394818000	-0.534853000	0.274115000
H	-6.855127000	-1.378076000	2.009916000
H	-4.661203000	-0.242034000	2.358358000
H	-7.898802000	1.671621000	-0.856969000
C	-5.127837000	0.032252000	-1.228368000
H	-5.048861000	0.979066000	-1.766446000
C	-6.360882000	-0.633355000	-1.176101000
H	-7.144217000	-0.300808000	-1.861614000
H	-6.380341000	-1.704286000	-0.966778000
C	-3.833111000	-0.566423000	-0.904126000
C	-3.805779000	-1.939166000	-0.258452000
H	-4.588698000	-2.058591000	0.503928000
H	-3.985315000	-2.704158000	-1.032969000
H	-2.819597000	-2.119617000	0.188551000
O	-2.784112000	0.019321000	-1.186530000
C	2.000396000	-1.072860000	0.220153000
C	2.917345000	-1.625451000	-0.724462000
C	4.259490000	-1.900280000	-0.324598000
C	4.678611000	-1.626286000	1.009987000
C	3.756566000	-1.084391000	1.951103000
C	2.413556000	-0.805522000	1.560110000
H	4.974296000	-2.284359000	-1.055870000
H	5.717407000	-1.795812000	1.301062000
H	4.087116000	-0.839528000	2.963034000
H	1.717473000	-0.357408000	2.271722000
I	-0.022535000	-0.661020000	-0.372611000
H	2.604015000	-1.805377000	-1.754655000
C	5.330328000	1.876300000	0.156541000
C	4.129250000	2.373907000	0.753931000
C	5.039732000	1.534964000	-1.202390000
H	6.303555000	1.801657000	0.641331000
C	3.096168000	2.340714000	-0.236240000
H	4.025952000	2.740828000	1.774791000
C	3.658673000	1.823153000	-1.444950000
H	5.753148000	1.157027000	-1.934579000
H	2.065536000	2.667311000	-0.100088000
H	3.133148000	1.694625000	-2.390913000

Tc	3.776039000	0.219034000	0.144502000

TS-Cat1-Re
***E* =** -1264.969496
***H* =** -1264.944254
***G* =** -1265.029746
N_{imag} = 1, -444 cm^{-1}
***SP* =** -1266. 3672878

C	-5.833233000	1.601795000	-0.453672000
C	-7.056973000	1.499769000	-1.329378000
C	-8.253909000	0.799964000	-0.625602000
C	-7.908201000	0.290816000	0.764451000
C	-7.032282000	1.048499000	1.552571000
C	-5.950381000	1.692423000	0.921851000
H	-9.081324000	1.519118000	-0.507876000
H	-7.340556000	2.534496000	-1.593285000
H	-6.805999000	1.015445000	-2.284326000
H	-4.900208000	1.901621000	-0.938546000
H	-8.705293000	-0.262536000	1.271395000
H	-7.039544000	0.939427000	2.641034000
H	-5.113710000	2.062659000	1.520608000
H	-8.651926000	-0.019371000	-1.243496000
C	-5.601873000	-0.887533000	-0.530927000
H	-5.703902000	-0.845566000	-1.617210000
C	-6.683396000	-1.321365000	0.249048000
H	-7.511888000	-1.813306000	-0.266467000
H	-6.496902000	-1.704107000	1.254017000
C	-4.216897000	-0.791337000	-0.070694000
C	-3.925083000	-0.935584000	1.411058000
H	-4.695946000	-0.468802000	2.039828000
H	-3.894494000	-2.008278000	1.667610000
H	-2.943929000	-0.497683000	1.636748000
O	-3.297750000	-0.635199000	-0.879552000
C	1.712729000	-1.225034000	-0.040285000
C	2.566971000	-1.433154000	-1.166922000
C	3.962589000	-1.659473000	-0.962651000
C	4.499268000	-1.676551000	0.359218000
C	3.642537000	-1.472282000	1.482238000
C	2.245280000	-1.245461000	1.286049000
H	4.623362000	-1.777090000	-1.823792000
H	5.572664000	-1.805871000	0.509562000
H	4.058312000	-1.449159000	2.491539000
H	1.594498000	-1.059224000	2.142002000
I	-0.397961000	-0.942639000	-0.342868000
H	2.162027000	-1.388763000	-2.179382000
C	4.869965000	1.984390000	-0.002236000
C	4.008320000	2.176594000	1.126033000
C	4.066138000	2.035276000	-1.186725000
H	5.951740000	1.860397000	0.032477000
C	2.671784000	2.347236000	0.637911000
H	4.320282000	2.222220000	2.168834000
C	2.707776000	2.259778000	-0.790480000
H	4.428639000	1.953499000	-2.210792000
H	1.787245000	2.539170000	1.244106000
H	1.854723000	2.370007000	-1.459103000
Re	3.374124000	0.249207000	0.065704000

TS-Cat1-Fe
***E* =** -2450.089744
***H* =** -2450.065273
***G* =** -2450.148059

N

C	5.152973000	-1.332972000	-0.113450000
C	6.317493000	-1.272599000	-1.067652000
C	7.426861000	-0.280671000	-0.618061000
C	7.061476000	0.490574000	0.640692000
C	6.320759000	-0.167878000	1.632618000
C	5.319056000	-1.074171000	1.234011000
H	8.350175000	-0.839564000	-0.394078000
H	6.734774000	-2.294729000	-1.114386000
H	5.966221000	-1.057606000	-2.087416000
H	4.253875000	-1.850803000	-0.458677000
H	7.800217000	1.230820000	0.964280000
H	6.348880000	0.190382000	2.665746000
H	4.561129000	-1.397128000	1.952780000
H	7.684717000	0.416856000	-1.429480000
C	4.606490000	1.073648000	-0.748443000
H	4.702640000	0.802156000	-1.801349000
C	5.640348000	1.785294000	-0.119774000
H	6.384326000	2.258843000	-0.765447000
H	5.423136000	2.350646000	0.788365000
C	3.267805000	0.883768000	-0.212652000
C	2.976882000	1.304406000	1.213726000
H	3.817412000	1.101360000	1.891318000
H	2.792963000	2.392131000	1.234457000
H	2.077137000	0.793525000	1.580433000
O	2.367526000	0.424190000	-0.932793000
C	-2.179502000	-0.997808000	0.079722000
C	-2.700665000	-0.936457000	1.398748000
C	-4.032863000	-1.339072000	1.663458000
C	-4.860394000	-1.797900000	0.611766000
C	-4.350371000	-1.850876000	-0.706827000
C	-3.018182000	-1.448608000	-0.973284000
H	-4.442542000	-1.213172000	2.666259000
H	-5.909088000	-2.027157000	0.801764000
H	-5.005377000	-2.121010000	-1.535815000
H	-2.663966000	-1.417447000	-2.004017000
I	-0.193713000	-0.323767000	-0.328997000
H	-2.100859000	-0.507486000	2.201824000
C	-5.668416000	1.316351000	0.117642000
C	-5.099400000	1.272672000	-1.191240000
C	-4.670059000	1.800170000	1.016585000
H	-6.672830000	0.995621000	0.388994000
C	-3.749586000	1.730109000	-1.101059000
H	-5.594044000	0.913429000	-2.092096000
C	-3.484532000	2.056039000	0.262861000
H	-4.779907000	1.914109000	2.093698000
H	-3.032078000	1.776184000	-1.918747000
H	-2.529533000	2.391641000	0.664220000
Fe	-4.019147000	0.064632000	0.095778000

TS-Cat1-Os
***E* =** -1277.229625
***H* =** -1277.204747
***G* =** -1277.289074
***N*$_{\mathbf{imag}}$ =** 1, -436 cm^{-1}
***SP* =** -1278.6320446

C	-5.651140000	1.656119000	-0.335267000
C	-6.816868000	1.618687000	-1.289380000
C	-8.059391000	0.886009000	-0.710305000
C	-7.804164000	0.271993000	0.657100000

C	-6.968218000	0.959281000	1.548771000
C	-5.845850000	1.635389000	1.033018000
H	-8.886024000	1.605054000	-0.588834000
H	-7.077359000	2.670920000	-1.503990000
H	-6.506727000	1.195731000	-2.255991000
H	-4.688165000	1.978842000	-0.740626000
H	-8.642114000	-0.293005000	1.077808000
H	-7.042676000	0.772806000	2.624029000
H	-5.044084000	1.949678000	1.706555000
H	-8.426406000	0.118755000	-1.408910000
C	-5.466251000	-0.872583000	-0.579787000
H	-5.520099000	-0.761389000	-1.664515000
C	-6.594850000	-1.317437000	0.127605000
H	-7.401076000	-1.769904000	-0.455425000
H	-6.462296000	-1.763708000	1.114860000
C	-4.113377000	-0.809867000	-0.050747000
C	-3.883666000	-1.025449000	1.431622000
H	-4.717118000	-0.665114000	2.048935000
H	-3.771309000	-2.106750000	1.620530000
H	-2.955874000	-0.525460000	1.739903000
O	-3.151910000	-0.626036000	-0.814206000
C	1.625860000	-1.244683000	-0.052497000
C	2.477067000	-1.411995000	-1.184987000
C	3.873342000	-1.634252000	-1.011497000
C	4.432489000	-1.679546000	0.295087000
C	3.592282000	-1.497002000	1.427921000
C	2.195475000	-1.274618000	1.255165000
H	4.519107000	-1.716544000	-1.886557000
H	5.508727000	-1.796607000	0.425432000
H	4.021968000	-1.475400000	2.430134000
H	1.568027000	-1.088712000	2.127468000
I	-0.475150000	-0.933845000	-0.320213000
H	2.066513000	-1.330718000	-2.192038000
C	4.803021000	1.925268000	0.005095000
C	3.958537000	2.099327000	1.150957000
C	3.980660000	2.003809000	-1.167247000
H	5.880498000	1.769288000	0.021998000
C	2.614641000	2.285682000	0.686373000
H	4.282173000	2.096731000	2.190577000
C	2.628268000	2.226733000	-0.745215000
H	4.323436000	1.916396000	-2.197020000
H	1.736579000	2.442867000	1.310764000
H	1.761868000	2.328398000	-1.397052000
Os	3.315835000	0.265062000	0.057713000

TS-Cat1-Co
***E* =** -2568.907581
***H* =** -2568.883257
***G* =** -2568.963117
N_{imag} = 1, -427 cm^{-1}
***SP* =** -2570.4789709

C	5.154056000	-1.339476000	0.109514000
C	6.210116000	-1.344214000	-0.962668000
C	7.295426000	-0.249806000	-0.766845000
C	7.031498000	0.650611000	0.430738000
C	6.415714000	0.097152000	1.563301000
C	5.422024000	-0.885750000	1.384483000
H	8.273591000	-0.727836000	-0.597163000
H	6.681476000	-2.343264000	-0.923373000
H	5.743565000	-1.286648000	-1.956625000
H	4.254280000	-1.932396000	-0.076774000

H	7.779810000	1.431980000	0.596657000
H	6.527692000	0.583474000	2.536443000
H	4.750783000	-1.135074000	2.210199000
H	7.409949000	0.358438000	-1.676957000
C	4.487058000	1.037336000	-0.805233000
H	4.564596000	0.695194000	-1.839365000
C	5.521372000	1.824714000	-0.268165000
H	6.192738000	2.303515000	-0.986308000
H	5.325207000	2.434328000	0.615857000
C	3.205406000	0.774401000	-0.200368000
C	2.913449000	1.200695000	1.221521000
H	3.811027000	1.433026000	1.806088000
H	2.272170000	2.097821000	1.195488000
H	2.351620000	0.404487000	1.730517000
O	2.304083000	0.227651000	-0.876704000
C	-2.125123000	-1.004336000	0.087150000
C	-2.697821000	-0.949440000	1.390278000
C	-4.038720000	-1.343283000	1.618118000
C	-4.845214000	-1.779505000	0.539720000
C	-4.301079000	-1.814211000	-0.766071000
C	-2.961697000	-1.414217000	-0.990390000
H	-4.473160000	-1.228156000	2.612290000
H	-5.900050000	-2.005205000	0.699741000
H	-4.938195000	-2.062124000	-1.616302000
H	-2.587261000	-1.364639000	-2.014228000
I	-0.104642000	-0.396362000	-0.272873000
H	-2.117176000	-0.541343000	2.219080000
C	-5.614219000	1.285267000	-0.427638000
C	-4.559894000	1.449488000	-1.379950000
C	-5.108664000	1.654098000	0.857185000
H	-6.606544000	0.886117000	-0.634999000
C	-3.404647000	1.923704000	-0.683786000
H	-4.608080000	1.196577000	-2.438645000
C	-3.743147000	2.050785000	0.697846000
H	-5.649863000	1.591447000	1.800429000
H	-2.418262000	2.089469000	-1.116360000
H	-3.063672000	2.340914000	1.498335000
Co	-3.999934000	0.111919000	0.071832000

TS-Cat1-Rh
E **=** -1296.862860
H **=** -1296.838060
G **=** -1296.920270
N_{imag} **=** 1, -434 cm^{-1}
SP **=** -1298.2825958

C	-4.961812000	1.691477000	-0.042689000
C	-6.035512000	1.891432000	-1.078433000
C	-7.365753000	1.165033000	-0.735590000
C	-7.281700000	0.327703000	0.531244000
C	-6.480277000	0.790754000	1.585778000
C	-5.272691000	1.448550000	1.280126000
H	-8.159733000	1.911418000	-0.571572000
H	-6.213351000	2.980993000	-1.132139000
H	-5.661267000	1.612800000	-2.074057000
H	-3.947535000	1.997507000	-0.313593000
H	-8.197975000	-0.210917000	0.792907000
H	-6.663396000	0.439382000	2.605280000
H	-4.514226000	1.577626000	2.056508000
H	-7.704490000	0.543824000	-1.578489000
C	-4.978928000	-0.825372000	-0.717959000
H	-4.984186000	-0.558551000	-1.776742000

C	-6.171416000	-1.261159000	-0.112963000
H	-6.971435000	-1.585017000	-0.784153000
H	-6.119334000	-1.837887000	0.812323000
C	-3.656777000	-0.897164000	-0.141251000
C	-3.472272000	-1.298944000	1.306031000
H	-4.365553000	-1.136882000	1.920965000
H	-3.218795000	-2.372004000	1.344931000
H	-2.630835000	-0.739459000	1.737911000
O	-2.655619000	-0.670382000	-0.855173000
C	1.958063000	-1.220486000	-0.021386000
C	2.841759000	-1.394195000	-1.129045000
C	4.231665000	-1.598837000	-0.928676000
C	4.769881000	-1.604636000	0.383805000
C	3.907842000	-1.410871000	1.493816000
C	2.518128000	-1.208702000	1.291366000
H	4.893203000	-1.697305000	-1.791469000
H	5.844980000	-1.712548000	0.535765000
H	4.319718000	-1.365583000	2.503720000
H	1.876613000	-1.013565000	2.153088000
I	-0.137067000	-0.942511000	-0.330257000
H	2.451746000	-1.341672000	-2.147519000
C	5.114755000	2.007895000	0.005611000
C	4.249355000	2.197224000	1.131613000
C	4.309048000	2.033804000	-1.179432000
H	6.191581000	1.844274000	0.045227000
C	2.909058000	2.348179000	0.641639000
H	4.553039000	2.206161000	2.178332000
C	2.946394000	2.248781000	-0.785989000
H	4.665825000	1.896904000	-2.200209000
H	2.015610000	2.487559000	1.250168000
H	2.085706000	2.293102000	-1.453688000
Rh	3.655350000	0.366960000	0.079240000

TS-Cat1-CO
***E* =** -1427.639587
***H* =** -1427.612547
***G* =** -1427.698454
N_{imag} = 1, -426 cm^{-1}
***SP* =** -1429.1791409

C	5.781324000	-1.315252000	-0.040715000
C	6.785459000	-1.052754000	-1.137117000
C	7.753423000	0.122845000	-0.814757000
C	7.394397000	0.869039000	0.459483000
C	6.873222000	0.134889000	1.540343000
C	6.010262000	-0.951565000	1.270484000
H	8.771294000	-0.270658000	-0.672746000
H	7.365885000	-1.988811000	-1.256135000
H	6.270400000	-0.918604000	-2.095774000
H	4.961523000	-2.002959000	-0.279455000
H	8.047859000	1.709864000	0.705695000
H	6.932720000	0.530670000	2.559713000
H	5.395039000	-1.374512000	2.070783000
H	7.810145000	0.822337000	-1.659801000
C	4.801161000	1.096223000	-0.709668000
H	4.891648000	0.874914000	-1.771492000
C	5.755192000	1.926964000	-0.093619000
H	6.355437000	2.550765000	-0.755508000
H	5.511860000	2.410768000	0.857468000
C	3.575818000	0.614758000	-0.117224000
C	3.280089000	0.847958000	1.345337000
H	2.541860000	1.668898000	1.430526000

H	2.814389000	-0.054131000	1.773273000
H	4.160641000	1.112609000	1.944716000
O	2.722614000	0.043792000	-0.842618000
C	-1.680562000	-1.107969000	0.166770000
C	-2.221737000	-0.876665000	1.478735000
C	-3.528075000	-1.306765000	1.814459000
C	-4.373526000	-1.887836000	0.832626000
C	-3.871792000	-2.076326000	-0.479601000
C	-2.553658000	-1.651131000	-0.823377000
H	-3.899953000	-1.136192000	2.827799000
H	-5.388294000	-2.192541000	1.086138000
H	-4.515684000	-2.504301000	-1.253411000
H	-2.206474000	-1.780578000	-1.848672000
I	0.341555000	-0.587627000	-0.269038000
H	-1.604791000	-0.396429000	2.242702000
Ru	-3.732487000	0.204790000	0.030285000
C	-4.941492000	0.559525000	-1.438080000
C	-2.633350000	1.717861000	-0.492943000
C	-4.796409000	1.361817000	1.169582000
O	-1.961700000	2.581948000	-0.788025000
O	-5.656289000	0.736680000	-2.297556000
O	-5.422993000	2.002414000	1.857741000

TS-Cat1-NCH
***E* =** -1367.982946
***H* =** -1367.953819
***G* =** -1368.045872
N_{imag} = 1, -430 cm^{-1}
***SP* =** -1369.4780345

C	-5.520725000	-1.322865000	-0.117516000
C	-6.627269000	-1.370266000	0.902325000
C	-7.734113000	-0.306758000	0.660763000
C	-7.438814000	0.608492000	-0.517995000
C	-6.761157000	0.075915000	-1.625164000
C	-5.748379000	-0.877592000	-1.404377000
H	-8.688023000	-0.812746000	0.440908000
H	-7.066358000	-2.382175000	0.832624000
H	-6.212196000	-1.306547000	1.918498000
H	-4.612775000	-1.889319000	0.107311000
H	-8.196664000	1.374306000	-0.711432000
H	-6.844596000	0.562013000	-2.601341000
H	-5.034343000	-1.105009000	-2.200043000
H	-7.911025000	0.291093000	1.567710000
C	-4.951270000	1.038622000	0.824936000
H	-5.051759000	0.681983000	1.852231000
C	-5.985160000	1.810343000	0.266064000
H	-6.695739000	2.257647000	0.966543000
H	-5.773995000	2.439413000	-0.600724000
C	-3.635718000	0.827906000	0.264265000
C	-3.313451000	1.286819000	-1.142487000
H	-4.199675000	1.484485000	-1.756642000
H	-2.718258000	2.213543000	-1.081032000
H	-2.694681000	0.526502000	-1.639842000
O	-2.742177000	0.302490000	0.960091000
C	1.683933000	-1.092651000	-0.096905000
C	2.210094000	-0.998723000	-1.435165000
C	3.508514000	-1.457707000	-1.725836000
C	4.340382000	-1.995897000	-0.689991000
C	3.841761000	-2.060803000	0.623304000
C	2.517713000	-1.597733000	0.926434000
H	3.908429000	-1.335071000	-2.734210000

H	5.365203000	-2.299535000	-0.907827000
H	4.488227000	-2.401409000	1.434887000
H	2.168329000	-1.613054000	1.960097000
I	-0.295704000	-0.412781000	0.329101000
H	1.610484000	-0.543705000	-2.225174000
Ru	3.662856000	0.057631000	-0.082694000
N	4.558932000	0.726436000	1.619806000
N	5.126382000	1.048626000	-1.096256000
N	2.641714000	1.821304000	-0.070174000
C	2.029531000	2.794096000	-0.051081000
C	5.956562000	1.599505000	-1.669877000
C	5.059794000	1.095912000	2.586290000
H	6.740471000	2.116016000	-2.212812000
H	5.532611000	1.440097000	3.499617000
H	1.447856000	3.708877000	-0.035451000

TS-Cat1-CNH
***E* =** -1368.006748
***H* =** -1367.977769
***G* =** -1368.069305
***N*$_{imag}$ =** 1, -426 cm^{-1}
***SP* =** -1369.4983437

C	-5.317643000	1.616366000	0.536575000
C	-6.316916000	2.161709000	-0.451289000
C	-7.615495000	1.309377000	-0.543147000
C	-7.566884000	0.046643000	0.307238000
C	-6.888698000	0.096585000	1.533858000
C	-5.715031000	0.865763000	1.629236000
H	-8.469400000	1.903313000	-0.176509000
H	-6.564505000	3.178608000	-0.102716000
H	-5.845707000	2.299825000	-1.436772000
H	-4.308030000	2.039732000	0.506725000
H	-8.457560000	-0.587144000	0.245274000
H	-7.126138000	-0.630861000	2.318677000
H	-5.030058000	0.717320000	2.469926000
H	-7.845717000	1.056160000	-1.589494000
C	-5.085473000	-0.445753000	-1.039550000
H	-5.005989000	0.221525000	-1.904350000
C	-6.295196000	-1.115519000	-0.794410000
H	-7.008169000	-1.163483000	-1.617753000
H	-6.287821000	-2.014718000	-0.170963000
C	-3.818377000	-0.725927000	-0.402902000
C	-3.759921000	-1.681001000	0.771965000
H	-4.684077000	-1.695287000	1.363685000
H	-3.591080000	-2.703217000	0.386490000
H	-2.917111000	-1.417290000	1.426143000
O	-2.769181000	-0.219937000	-0.848142000
C	1.804046000	-1.162330000	0.089098000
C	2.666115000	-1.522530000	-0.996483000
C	4.008928000	-1.867526000	-0.755391000
C	4.545390000	-1.835358000	0.562575000
C	3.712068000	-1.445189000	1.627825000
C	2.348894000	-1.096133000	1.396071000
H	4.654669000	-2.132620000	-1.599945000
H	5.590727000	-2.089930000	0.741369000
H	4.112634000	-1.380486000	2.639672000
H	1.719665000	-0.791261000	2.239198000
I	-0.260146000	-0.745375000	-0.266164000
H	2.278206000	-1.536649000	-2.015150000
Ru	3.709011000	0.302831000	0.066432000
C	4.328789000	1.536057000	1.437650000

C	5.142779000	0.934714000	-1.091443000
C	2.579105000	1.724472000	-0.639548000
N	1.873366000	2.544041000	-1.063344000
N	6.001174000	1.289761000	-1.786542000
N	4.695548000	2.251422000	2.278160000
H	6.748378000	1.591001000	-2.396143000
H	5.012494000	2.873513000	3.008158000
H	1.239806000	3.238655000	-1.432180000

Adduct
***E* =** -464.169098
***H* =** -464.158499
***G* =** -464.204323
N_{imag} = 0
***SP* =** -464.9029238

C	-0.272047000	0.876385000	0.721845000
C	-1.629139000	1.451039000	0.232874000
C	-2.392531000	0.359525000	-0.564335000
C	-1.559127000	-0.950453000	-0.564968000
C	-1.239536000	-1.311390000	0.866806000
C	-0.559766000	-0.364901000	1.533435000
H	-3.381549000	0.166857000	-0.121797000
H	-2.210419000	1.781012000	1.107211000
H	-1.444343000	2.342537000	-0.387402000
H	0.286633000	1.634187000	1.288579000
H	-2.103832000	-1.753331000	-1.082817000
H	-1.535278000	-2.270325000	1.301412000
H	-0.237657000	-0.459990000	2.573879000
H	-2.563139000	0.677264000	-1.605786000
C	0.542976000	0.479679000	-0.565563000
H	0.579531000	1.382343000	-1.194559000
C	-0.218054000	-0.660174000	-1.295007000
H	-0.423136000	-0.367148000	-2.336699000
H	0.373996000	-1.584521000	-1.340008000
C	1.999763000	0.231323000	-0.176692000
C	2.469106000	-1.176329000	0.119322000
H	1.734310000	-1.718998000	0.732832000
H	2.577861000	-1.736093000	-0.824849000
H	3.444600000	-1.137462000	0.621725000
O	2.756568000	1.179431000	-0.072253000

Adduct-cat1
***E* =** -1281.509189
***H* =** -1281.485249
***G* =** -1281.567430
N_{imag} = 0
***SP* =** -1282.9191293

C	-5.466304000	1.205645000	0.465234000
C	-6.609934000	2.074706000	-0.126161000
C	-7.812166000	1.162864000	-0.489938000
C	-7.486043000	-0.300875000	-0.086710000
C	-7.082441000	-0.315818000	1.368589000
C	-6.022658000	0.457451000	1.653268000
H	-8.726365000	1.492031000	0.026228000
H	-6.898400000	2.831065000	0.619072000
H	-6.237127000	2.616554000	-1.009783000
H	-4.601067000	1.831862000	0.724338000
H	-8.346540000	-0.956041000	-0.284656000
H	-7.613195000	-0.919357000	2.109937000
H	-5.589392000	0.560523000	2.651616000
H	-8.022776000	1.198995000	-1.571053000

C	-5.059934000	0.182410000	-0.665013000
H	-4.843538000	0.786031000	-1.558875000
C	-6.262714000	-0.764732000	-0.925277000
H	-6.521415000	-0.751987000	-1.995450000
H	-6.025800000	-1.806913000	-0.670449000
C	-3.729015000	-0.461947000	-0.310088000
C	-3.696686000	-1.734166000	0.498249000
H	-4.486125000	-1.740256000	1.262961000
H	-3.890054000	-2.587099000	-0.174448000
H	-2.708101000	-1.869399000	0.955840000
O	-2.697976000	0.091553000	-0.669080000
C	2.065418000	-1.123398000	-0.015516000
C	2.889216000	-1.358144000	-1.154151000
C	4.265050000	-1.676254000	-0.987936000
C	4.828171000	-1.753502000	0.313402000
C	4.011573000	-1.514309000	1.450476000
C	2.635103000	-1.195729000	1.288585000
H	4.896712000	-1.812131000	-1.867289000
H	5.894531000	-1.947955000	0.437005000
H	4.448122000	-1.525840000	2.450516000
H	2.026476000	-0.968672000	2.165065000
I	0.008344000	-0.624386000	-0.264999000
H	2.476665000	-1.255735000	-2.158933000
C	5.422653000	1.778692000	0.080798000
C	4.539731000	2.016596000	1.182884000
C	4.662583000	1.892186000	-1.127620000
H	6.485381000	1.549912000	0.149831000
C	3.234023000	2.278971000	0.654935000
H	4.812836000	2.001019000	2.237214000
C	3.310172000	2.202417000	-0.772134000
H	5.045572000	1.764705000	-2.139282000
H	2.337361000	2.490865000	1.236000000
H	2.481434000	2.344602000	-1.464699000
Ru	3.839355000	0.243519000	0.079355000

Adduct-cat2
***E* =** -993.368343
***H* =** -993.349640
***G* =** -993.419683
N_{imag} = 0
***SP* =** -994.454756

C	-2.379742000	0.563351000	-0.801603000
C	-3.342989000	1.444022000	-1.641018000
C	-4.516100000	1.925337000	-0.745998000
C	-4.321899000	1.368966000	0.689935000
C	-2.953299000	1.784687000	1.177694000
C	-1.941773000	1.360416000	0.403752000
H	-4.561537000	3.024393000	-0.711965000
H	-2.779285000	2.297700000	-2.046741000
H	-3.709968000	0.861392000	-2.501035000
H	-1.532785000	0.220416000	-1.411232000
H	-5.123657000	1.725600000	1.352882000
H	-2.818457000	2.373313000	2.089388000
H	-0.885440000	1.551866000	0.609651000
H	-5.484164000	1.575909000	-1.140547000
C	-3.207458000	-0.685510000	-0.313488000
H	-3.615233000	-1.157303000	-1.220827000
C	-4.353247000	-0.183325000	0.605428000
H	-5.325757000	-0.506644000	0.202186000
H	-4.279862000	-0.600777000	1.618700000
C	-2.231637000	-1.715428000	0.248662000

C	-2.017280000	-1.820125000	1.739967000
H	-1.888847000	-0.820839000	2.182963000
H	-2.904281000	-2.272183000	2.214460000
H	-1.140432000	-2.448595000	1.941993000
O	-1.592458000	-2.406129000	-0.528214000
C	3.081763000	0.212375000	-0.011619000
C	4.359892000	-0.354606000	-0.074919000
C	5.480852000	0.467798000	0.087862000
C	5.327154000	1.839947000	0.311201000
C	4.044250000	2.394028000	0.371618000
C	2.914025000	1.584396000	0.210297000
H	6.479757000	0.026790000	0.038786000
H	6.205950000	2.476709000	0.437631000
H	3.914878000	3.465415000	0.544966000
H	1.915433000	2.023199000	0.256322000
I	1.360978000	-1.023783000	-0.255272000
H	4.486456000	-1.424796000	-0.249065000
